# Molecular basis for DNA repair synthesis on short gaps by mycobacterial Primase-Polymerase C

**DOI:** 10.1038/s41467-020-18012-8

**Published:** 2020-08-21

**Authors:** Nigel C. Brissett, Katerina Zabrady, Przemysław Płociński, Julie Bianchi, Małgorzata Korycka-Machała, Anna Brzostek, Jarosław Dziadek, Aidan J. Doherty

**Affiliations:** 1 Genome Damage and Stability Centre, School of Life Sciences, University of Sussex, Brighton, BN1 9RQ UK; 2 Institute of Medical Biology, Polish Academy of Sciences, Lodowa 106, 93-232 Lodz, Poland; 3 Present Address: School of Pharmacy and Biomolecular Sciences, University of Brighton, Brighton, BN2 4GJ UK; 4 Present Address: Department of Oncology-Pathology, BioClinicum, Karolinska University Hospital, Stockholm, Sweden

**Keywords:** Enzyme mechanisms, X-ray crystallography, Bacteria, DNA synthesis

## Abstract

Cells utilise specialized polymerases from the Primase-Polymerase (Prim-Pol) superfamily to maintain genome stability. Prim-Pol’s function in genome maintenance pathways including replication, repair and damage tolerance. Mycobacteria contain multiple Prim-Pols required for lesion repair, including Prim-PolC that performs short gap repair synthesis during excision repair. To understand the molecular basis of Prim-PolC’s gap recognition and synthesis activities, we elucidated crystal structures of pre- and post-catalytic complexes bound to gapped DNA substrates. These intermediates explain its binding preference for short gaps and reveal a distinctive *modus operandi* called Synthesis-dependent Template Displacement (STD). This mechanism enables Prim-PolC to couple primer extension with template base dislocation, ensuring that the unpaired templating bases in the gap are ushered into the active site in an ordered manner. Insights provided by these structures establishes the molecular basis of Prim-PolC’s gap recognition and extension activities, while also illuminating the mechanisms of primer extension utilised by closely related Prim-Pols.

## Abstract (web summary)

Mycobacteria Prim-PolC performs short gap synthesis following removal of lesions during excision repair. Here the authors resolve crystal structures of pre- and post-catalytic Prim-PolC complexes bound to gapped DNA substrates to define its mechanism.

## Introduction

Cells contain diverse DNA polymerases critical for replication and repair mechanisms that maintain genome stability^1^. They also contain non-canonical polymerases called primases that undertake primer synthesis during replication. However, members of the Primase–Polymerase (Prim–Pol) superfamily, previously called archaeo-eukaryotic primases (AEPs), also play diverse roles in DNA metabolism, including repair and repriming^2^. For example, Prim–PolD (PolDom/LigD Pol) is required for the repair of double-strand breaks (DSBs) in many prokaryotes^3^. These bespoke repair polymerases possess a wide range of synthesis activities^4^, and also promote break synapsis via microhomology-mediated end joining (MMEJ) to facilitate efficient repair of DSBs by the non-homologous end-joining (NHEJ) pathway^5,6^.

Although Prim–PolD’s roles in NHEJ repair are well established, particularly in mycobacteria, the biological functions of closely related paralogues have remained unclear. Recently, it was reported that a closely related enzyme called Prim–PolC, operonically associated with ligase C (LigC), is specifically involved in LigC-dependent repair of short DNA-gapped intermediates produced during excision-repair processing of lesions in mycobacteria^7^, further expanding the repertoire of pathways in which these diverse replicases operate. A notable feature of Prim–PolC is its favourable activity on short DNA gaps of 1–3 nucleotides, which it preferentially fills in with ribonucleotides. Although the crystal structure of the apo Prim–PolC showed that it is remarkably similar to Prim–PolD, it contains a unique C-terminal extension called Loop 3, proposed to be potentially involved in gap recognition^7^.

To understand the molecular basis for its gap-binding and synthesis activities, we elucidated the crystal structures of pre- and post-catalytic intermediates of Prim–PolC bound to DNA substrates containing a two-nucleotide gap. Based on these structures, in conjunction with supporting biochemical analysis, we describe the structural elements, conformational steps and the underlying catalytic mechanism Prim–PolC employs to simultaneously engage with both sides of a gap, whilst extending the 3′ primer strand to fill in short-gapped DNA intermediates with ribonucleotides during excision repair.

## Results

### Overall structure of a ternary Prim–PolC–DNA–NTP complex

**Fig. 1:**
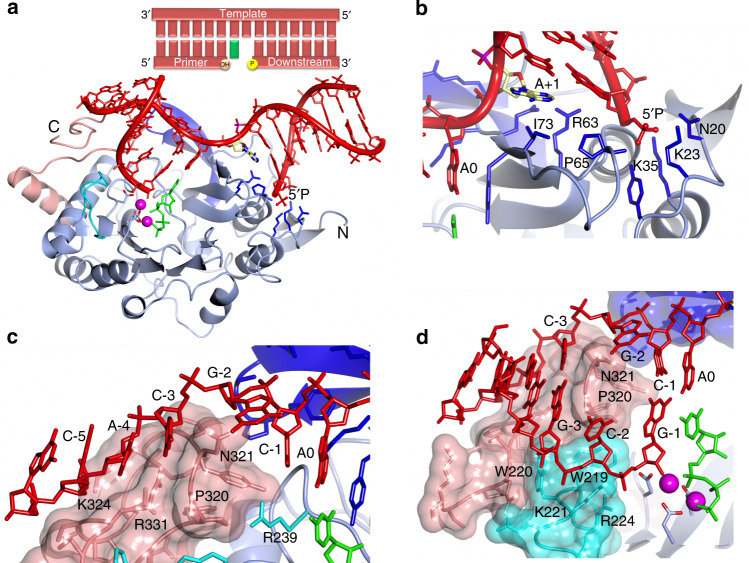
Crystal structure of a ternary Prim–PolC–DNA–NTP complex. **a** Schematic of the 2-nt gapped DNA substrate found in the ternary complex (top panel). The bottom panel shows a ribbon-diagram representation of the Prim–PolC–DNA–NTP complex (PDBID: 6SA0). The catalytic core is coloured sky blue, with the conserved loop structures from this family coloured dark blue, cyan and pink for Loops 1–3, respectively. The catalytic aspartate side chains are represented with the carboxylic oxygens shown in red and bound manganese ions depicted as magenta spheres. The incoming nucleotide, rUTP (UpNHpp), is coloured green. Side chains involved in forming the 5′ phosphate-binding pocket are depicted in dark blue. **b** Molecular interactions of the N-terminal region of Prim–PolC with the 5′-phosphate moiety (downstream stand) and the dsDNA/ssDNA junction. **c** Details of the interactions of Loop 3 with upstream template DNA. The primer strand is removed for clarity. **d** A view of the solvent-accessible surfaces of Loops 2 (cyan) and 3 (salmon) interacting with the upstream DNA duplex. N321 is shown to interdigitate into the DNA duplex and, with P320, cause the DNA to unpair at the −2 position. Loop 2 supports the primer strand of the upstream duplex.

Previously, we reported that Prim–PolC preferentially binds to and fills in short DNA gaps with ribonucleotides during excision repair^7^. To understand the molecular basis for substrate recognition and synthesis, we crystallised Prim–PolC (Prim–PolC_4–336_) in complex with dsDNA containing a two-nucleotide (2-nt) gap (Fig. 1a). To determine the structure of a pre-catalytic ternary complex of the enzyme bound to the DNA, a non-hydrolysable incoming UTP ribonucleotide (UpNHpp) and manganese ions were also included. Crystals were obtained that diffracted to a resolution of 2.2 Å and contained two Prim–PolC–DNA-bound complexes per asymmetric unit. The structure was solved by molecular replacement using the *apo* Prim–PolC structure (see ‘Methods’) and refined at 2.2-Å resolution (Table 1). Comparing the current structure with the previously elucidated Apo crystal structure (PDBID: 5Op0) showed that the overall core fold of the enzyme is maintained, although the root-mean-squared deviation of atomic positions (RMSD) was 1.72 Å (over 327 Cα positions). This high RMSD value is mainly due to Loop 3 adopting a different conformation compared with the Apo structure (Supplementary Fig. 1), described below.

**Table 1:** Data collection and refinement statistics (molecular replacement).

	*Msm* Prim–PolC ternary complex (PDB: 6SA0)	*Msm* Prim–PolC post-catalytic pre-ternary complex (PDB: 6SA1)
*Data collection*		
Space group	P 3_1_ 2 1	P 4 2_1_ 2
Cell dimensions		
* a*, *b*, *c* (Å)	136.06, 136.06, 133.48	198.25, 198.25, 85.30
* α*, *β*, *γ* (°)	90, 90, 120	90, 90, 90
Resolution (Å)	42.25 (2.21)^*^	70.09 (2.01)^*^
*R*_sym_ or *R*_merge_	0.102 (1.298)	0.047 (1.212)
*I*/*σI*	8.53 (1.66)	20.05 (1.33)
Completeness (%)	98.89 (97.98)	99.91 (99.87)
Redundancy	6.4 (6.4)	6.4 (5.8)
cc^*^	0.999 (0.887)	1.000 (0.919)
*cc1/2*	0.996 (0.65)	0.999 (0.778)
*Refinement*		
Resolution (Å)	42.25 (2.21)	70.09 (2.01)
No. of reflections	71044 (6969)	112731 (11116)
*R*_work_/*R*_free_	0.1746/0.2069	0.1845/0.2042
No. of atoms	6955	6755
Protein	6418	6312
Ligands	79	134
Water	458	362
*B* factors		
Protein	50.98	73.63
Ligands	47.68	103.54
Water	50.41	57.53
R.M.S. deviations		
Bond lengths (Å)	0.013	0.010
Bond angles (°)	1.31	1.01

^*^From 1 crystal. *Values in parentheses are for the highest-resolution shell.

Briefly, the elucidated structure shows Prim–PolC bound to a 2-nt gapped DNA substrate (Fig. 1a) in the act of incorporating the appropriate ribonucleotide (UTP) opposite the templating base (A at position 0) in the single-stranded gap (Fig. 1b–d). The DNA substrate consists of a 15-nt template (T) strand annealed to a 6-nt primer (P) strand and a 7-nt downstream (D) strand, with a central 2-nt gap (see ‘Methods’). Residues in Loops 1 and 3 are directly involved in binding to and stabilising the DNA template strand, including the 2-nt gap. Residues in Loop 2 contact and support the incoming primer strand, which is docked into the active site of the enzyme, positioning the incoming 3′-OH within the attacking distance of the ribonucleotide analogue (UpNHpp) and metal ions, thus forming a pre-turnover ternary complex (Fig. 1a, d).

### Interactions within the Prim–PolC–DNA ternary complex

Previously, we reported that the presence of a terminal 5′ phosphate on gap substrates enhances Prim–PolC’s DNA binding and repair-synthesis activities^7^. The structure reveals that Prim–PolC contains a conserved phosphate-binding pocket, consisting of N20, K23, Y25, K35 and P65, that engages with the 5′ phosphate on the downstream strand, positioning the N-terminal region of the enzyme on the 5′ side of the gap (Fig. 1b). Additional contacts between R63, P65 and the exposed terminal nucleotide base (D/G + 2) at the dsDNA interface also aid in docking the enzyme onto the 5′ side of the gapped substrate (Fig. 1b). Binding of Prim–PolC to DNA induces a major splaying of the template strand by 97.3°. A hydrophobic molecular wedge-like structure, formed by I73 and Y74, analogous to F63 and F64 of Prim–PolD^5,6^, is placed between bases A0 and A + 1 of the templating strand, inducing a pronounced splaying of the DNA. Notably, this insertion of residues between the unpaired bases of the 2-nt gap appears to facilitate the selective incorporation of a nucleotide at the 0-template position, the unpaired position next to the upstream duplex side of the wedge, proximal to the active site. The second unpaired base (A, position +1) is located on the wedge distal to the active site between the N-terminal region of the protein and the downstream duplex, where the downstream duplex would be expected to sit (compared with previous Prim–PolD/DNA structures). The accommodation of the second unpaired base on I73 and Q75 (base and sugar/backbone contacts, respectively) results in the downstream duplex being offset from the expected axis observed previously in Prim–PolD/DNA structures^5,6,8^. This is an example of a hitherto undescribed mechanism for nucleotide incorporation into a gap, without requirement for template scrunching, which we term synthesis-dependent template displacement (STD).

Prim–PolC’s highly conserved Loop 1 also assisted in orienting the templating strand through explicit interactions between the phosphate group of T/G-2 and S95 and R97 (Supplementary Fig. 2a). Together with R77, Loop 1 plays a pivotal role in maintaining the templating strand orientation that allows placement of the unpaired base (A0) into the active site, for subsequent pairing with the incoming UTP nucleotide. The role and orientation of Loop 1 is similar to that described previously in Prim–PolD/DNA structures^5,6,8^. The process of STD results in the placement of the templating base (A0) into the active site.

Continuing in the 3′ direction along the DNA template strand, further interactions are formed with Loop 3 (Prim–PolC_313–333_) (Fig. 1c), which undergoes the greatest movement from the Apo structure, with a significant structural rearrangement resulting in a RMSD of 4.47 Å (over 20 Cα positions). The templating strand sits on Loop 3 and N321 interdigitates into the upstream duplex region, flipping out a guanine base (T/G-2) from the templating strand, and causes it to adopt an extra-helical conformation (Fig. 1d). N321 appears to act as a molecular locating pin that stops the upstream duplex from slipping back and forth. The side chain of N321 hydrogen bonds to T/G-2 and T/C-3. Further non-bonded interactions between P320 and the base of P/C-2, on the primer strand, provide a surface that stabilises the upstream DNA duplex. P320 also makes Van der Waals interactions to the C-2 atom of the base in the primer strand P/C-2 (Fig. 1d). This interaction fully disrupts the base pairing that would have occurred at this position. Furthermore, K324 makes contacts with T/A-4 and T/C-5. R331 also hydrogen bonds with T/C-5. Together, these Loop 3 contacts play a major role in locking the template strand and upstream duplex in place (Fig. 1d). Without these upstream interactions, the primer strand cannot be docked in a suitable orientation to allow for catalytic extension. The observed interactions suggest that the role of Loop 3 is to ensure that Prim–PolC binds specifically to short-gapped substrates. Loop 2 also plays a key role in docking onto the incoming primer strand via non-bonded interactions with W219, W220, K221 and the backbone atoms of the DNA at P/C-2. The incoming base of the primer strand (P/G-1) is stabilised by R224 via hydrogen bonding (Fig. 1d).

### Configuring the active site for gap-filling synthesis

**Fig. 2:**
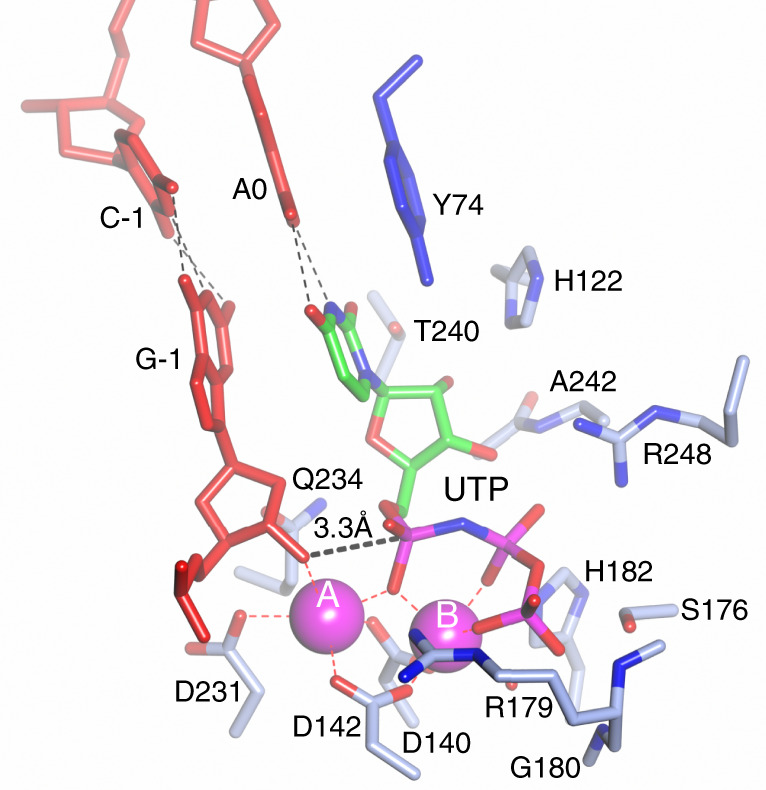
Active-site geometry of the Prim–PolC–DNA ternary complex. Molecular details of the docking of the 3′-terminal nucleotide of the incoming primer strand as the enzyme positions the 3′-OH moiety to attack the α-phosphate of the bound non-hydrolysable UTP analogue, UpNHpp. Hydrogen bonds are depicted in black, and dative bonding of the catalytic manganese ions (magenta spheres) is depicted in red. R179 appears to act as a gating side chain and is evidently in a closed position.

Prim–PolC has evidently evolved to bind short-gapped DNA intermediates with the purpose of correctly positioning the 3′-OH of the primer strand into its active site in readiness for extension. The enzyme also has a preference for insertion of ribonucleotides over deoxyribonucleotides^7^. In this ternary complex structure, we observe that the 3′ end of the primer strand adopts a C-3′-endo conformation, and is docked in the active site with the incoming 3′-OH of G-1 positioned only 3.3 Å away from the α-phosphate of the incoming base, and almost in line with the α–β-bond position of the ribonucleotide analogue (UpNHpp). Because of the high activation energy of the leaving group (NH_2_pp), a stabilised ternary complex is formed that cannot be turned over. The 3′-OH of the primer strand also interacts with the Mn^2+^ ion in the A position of the active site at a distance of 2.8 Å. This 3′-OH splits the axial position for interaction with the A-site metal ion with the α-phosphate of UpNHpp (Fig. 2). The A- and B-site Mn^2+^ ions are chelated by the carboxylate groups of D231, D140 and D142 for site A and D140 and D142 for site B. Further ligands for the B- site Mn^2+^ are provided by the α-, β- and γ-phosphate oxygens of UpNHpp. Both A and B (Mn^2+^ sites) possess octahedral coordination geometry, which is the optimised reactant-state geometry observed in other polymerase ternary structures^9–11^. The two-site octahedral arrangement allows the reactants to be precisely aligned to allow chemistry to commence. Under ideal conditions, the A-site Mn^2+^ coordinates and deprotonates the incoming 3′-OH allowing it to act as a nucleophile that attacks the α-phosphate, via an S_N_2 mechanism, along the line of the α–β-phosphate bond. A pentavalent transition phosphate intermediate is formed, which decomposes via an inversion of the phosphate centre and concomitant loss of inorganic phosphate (PPi) to yield an extended primer strand. The incoming ribonucleotide, UpNHpp, adopts an anti-conformation with its sugar observed to have a C-3′-endo pucker (Fig. 2). UpNHpp base pairs in a normal Watson–Crick (W–C) fashion with the T/A0 base in the gap on the template^12^ strand, and is further stabilised in the active site by a network of hydrogen and non-bonded interactions between H122, Q234, T240, A242 and the ribobase of UpNHpp (Fig. 2). The 2′OH of the ribobase hydrogen bonds with H122, T240 and A242, thus stabilising and orientating the nucleotide in an optimum conformation for transfer. This likely accounts for the enzyme’s ability to discriminate between ribo- and deoxyribonucleotides^7^. The phosphate tail of UpNHpp interacts with S176, R179, G180, H182 and R248 via hydrogen bonds (Fig. 2), in addition to the interactions already characterised.

### Base extrusion from the template strand occurs in solution

**Fig. 3:**
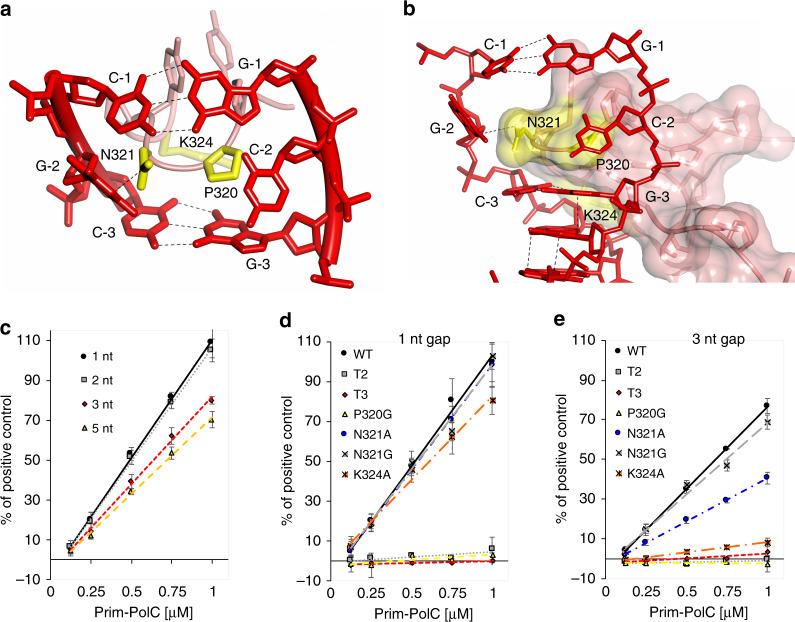
Loop 3 interactions with DNA are requisite for gap filling. **a**, **b** Schematic solvent-accessible surface representations of Loop 3 interacting with template strand DNA, shown in two orientations. Residues that interact are highlighted in yellow, and hydrogen bonds are depicted in black (hatched line). See also Supplementary Fig. 2b and c. **c** Flipping activity of Prim–PolC on different short-gap substrates examined using a 2-aminopurine (2-AP)-based assay. The ability of Prim–PolC to flip the −2 primer base (2-AP) was measured as an increase in 2-AP fluorescence intensity when the −2 base was unpaired. Each reaction contained 0, 0.125, 0.5, 0.75 or 1 µM Prim–PolC (WT), 1 µM substrate, 5 mM Ca^2+^ ions and 250 µM ATP. **d**, **e** Flipping activity of Prim–PolC variants. Quantification of primer −2 base flipping by Prim–PolC variants on 1-nt (**d**) or 3-nt gap (**e**) substrates under the same conditions as stated in (**c**). Data shown (**c**, **d** and **e**) are representative of the mean of at least three individual experiments, and error bars show the standard deviation.

The ternary complex structure reveals that Prim–PolC’s conserved Loop 3 (L3) makes extensive contacts with the upstream DNA, interdigitating with the upstream dsDNA inducing a base to be extruded, and it also makes additional contacts that may assist in docking the primer strand into the active site (Fig. 3a, b). To examine if base dislocation occurs in solution, we introduced 2-aminopurine (2-AP) into the primer at the −2-nt position (displaced base). 2-AP base fluorescence increases when it is unpaired, thus allowing us to monitor its mobility under a variety of experimental conditions. To trap Prim–PolC bound to DNA, forming ternary complexes in solution to increase the chances of observing this base-flipping phenomenon, assays were set up in the presence of an incoming base (ATP) and inhibitory calcium ions to prevent turnover. Next, we measured 2-AP fluorescence on a range of gapped substrates (Supplementary Fig. 3a), and observed significant increases in fluorescence on 1- and 2-nt gapped substrates, less so on 3-nt and even less on 5-nt gaps (Fig. 3c). These data also correlate with the Prim–Pol’s gap-filling efficiency observed on various gapped substrates (Supplementary Fig. 3b–d). Notably, no fluorescence changes were observed when assays were performed in the absence of ATP or calcium (Supplementary Fig. 4). Together, these findings establish that the −2-nt base is extruded from the template strand during ternary complex formation in solution.

To examine if specific residues are required for base dislocation on the template strand, we measured 2-AP fluorescence changes on a range of gapped substrates in the presence of WT, L3 mutants and truncations (T2 (Prim–PolC_1-312_) and T3 (Prim–PolC_1-319_)) of Prim–PolC (Supplementary Fig. 5). As expected, removal of all or part of L3 resulted in no base dislocation activity (Fig. 3d, e). For 1-nt gaps, N321 mutants exhibited similar fluorescence to WT, with K324A having a slightly reduced 2-AP signal. However, P320G elicited no increase in fluorescence, indicating that it is requisite for 2-AP base displacement (Fig. 3d). For 3-nt gaps, N321G exhibited similar fluorescence to WT, with N321A having a more reduced 2-AP signal, and K324A promoted only a very modest increase in fluorescence. P320G also exhibited no fluorescence increase on 3-nt gaps, confirming that P320 is critical for L3’s ability to induce base displacement (Fig. 3e). These results imply that substrates with gaps larger than 2 nt are not optimally dislocated. Thus, for 3-nt gaps, mutating N321 and K324 has more of an impact on base displacement. L3 evolved to deal with an optimal gap size of 1–2 nt, with the residues adapting to maintain some activity for larger gaps. Residues N321 and K324 have become more critical in maintaining contacts within the upstream strand of DNA during substrate binding.

### Loop 3 is required for efficient short gap-filling synthesis

**Fig. 4:**
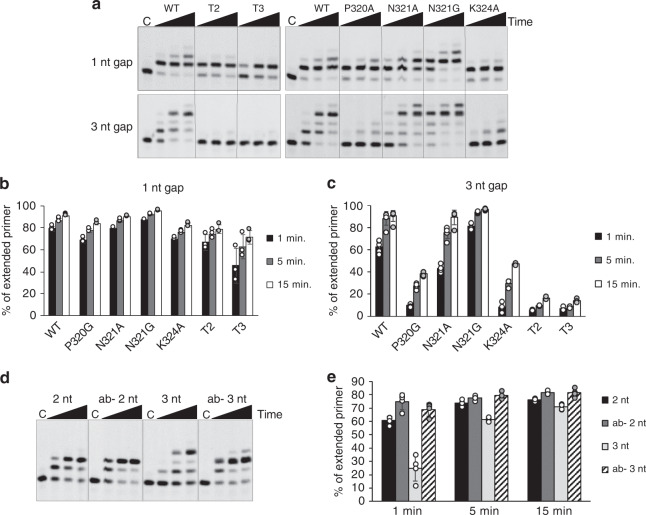
Gap-filling activities of Loop 3 mutants. **a** Short gap-filling activities of selected Prim–PolC mutations. In primer extension assays, 30 nM of 5′-fluorescein-labelled 36-mer containing a single-stranded 1-nt gap (top) or 3-nt gap (bottom) (with phosphorylation of the 5′ end of the downstream strand) was extended by Prim–PolC (300 nM) in the presence of a 250 μM rNTP mix for 1, 5 and 15 min at 37 °C. Control (C) lane contains no protein. **b**, **c** Quantification of the gap-filling assay (quantification of panel **a**). **d** Gap-filling activity of Prim–PolC (WT) on an abasic (template −2 position) 3-nt gap substrate. In primer extension assays, 30 nM of 5′-fluorescein-labelled 36-mer containing different single-stranded gaps, with phosphorylation of the 5′ end, was extended by Prim–PolC (30 nM) in the presence of a 250 μM rNTP mix for 1, 5 and 15 min at 37 °C. Control (C) lane contains no protein. **e** Quantification of Prim–PolC primer extension on an abasic 3-nt gap substrate (quantification of panel **e**). Data shown (**b**, **c** and **e**) are representative of the mean of at least three individual experiments, and error bars show the standard deviation, and dotplots show the corresponding data points.

To study the functional importance of Prim–PolC’s Loop 3 in gap-filling synthesis, we performed primer-template-extension assays using gapped substrates (1- or 3-nt gap). Although both truncations maintained their ability to fill in 1-nt gaps, similar to WT Prim–PolC, both L3 mutants were unable to fill in 3-nt gapped substrates, implicating this structural element in gap-repair synthesis on longer gaps. To determine the importance of specific L3 residues in gap-repair synthesis, we mutated three residues implicated in primer-template binding, including P320 and N321 that contact the upstream primer and template DNA, respectively, and K324 that interacts with the template strand (Supplementary Fig. 5). Four mutants (P320G, N321A, N321G and K324A) were purified to homogeneity, and thermal heat denaturation assays confirmed that these mutations did not significantly alter the stability of these proteins (Supplementary Fig. 6a, b). Their relative extension activities were then measured using gap-filling assays, and all point mutants were able to fill in 1-nt gapped substrates, comparable to WT and T2/T3 mutants. On 3-nt gaps, N321A/G performed almost as well as WT, in terms of total turnover, but produced more strand-displacement products than WT enzyme (Fig. 4a–c). However, P320G and K324A showed only limited and incomplete gap-filling synthesis on 3-nt substrates, indicating that these residues are requisite for filling longer gaps (>1 nt).

Given that Prim–PolC appears to invoke a base-flipping mechanism to promote gap filling, we posited what would happen if the base that is displaced was removed so that it no longer needs to be dislocated? To address this question, we replaced the base at the −2 position on the template strand with an abasic site (Ab) to act as a mimetic of this base-extruded intermediate. We next measured Prim–PolC’s gap-filling activities on Ab-modified or -unmodified 2- and 3-nt gapped substrates (Supplementary Fig. 6c). Notably, we observed that primer extension was more rapid (see 1-min time point—Fig. 4d, e) when the Ab site (base extruded) was present, but also limited to just inserting two bases, not three, into the 3-nt gapped substrate (Fig. 4d). This experiment suggests the way Prim–PolC interacts with the substrate has changed, which can be explained by holding it onto the Ab substrate and not releasing it before cycling to the next nucleotidyl transfer step. This finding supports the notion that the maximum gap size the enzyme can comfortably accommodate without having to seriously distort the substrate is 2 nt. This is further evidenced by the apparent rate of insertion of the first base dropping after the gap size increases (Supplementary Fig. 3d). Normally, we regard these repair enzymes as distributive in action, but the observed increase in enzyme rate points to the enzyme acting in a more processive manner.

In summary, these findings suggest that the template DNA strand needs to be properly aligned via interactions with L3; otherwise, it affects the orientation of the region upstream of the gap, thus preventing correct docking/orientation of the incoming primer strand in the active site. Removal of K324A has a major effect on turnover as this residue not only interacts with the template strand at A-4 and C-5, but is also responsible for maintaining the conformation of that region of L3 via side chain–backbone interactions. The unusual activity of T2 on the 1-nt gap may be due to the intrinsic helical structure of the DNA itself, allowing for proper substrate interaction without the blocking effect of L3. The N321A mutation suggests that removing this molecular pin from L3 allows increased freedom of movement of the upstream DNA, allowing more strand displacement synthesis to occur during extension. This suggests that another role of L3 may be to prevent displacement synthesis, an activity that Prim–PolD possesses, by preventing excessive movement of the templating strand. L3 effectively holds the upstream DNA in place and acts as a physical ruler judging what size gaps the enzyme will insert across, and preventing it from performing repair synthesis on longer gaps.

### Structures of post-catalytic Prim–PolC complexes

To elucidate the structural transitions that occur during Prim–PolC’s catalytic cycle, we next crystallised a post-catalytic 1-nt gap ternary complex by mixing Prim–PolC, 2-nt gap DNA, Mn^2+^ and a 3′-deoxy chain-terminating ribonucleotide, 3′-dUTP (3′-dUTP is UTP lacking a 3′-hydroxyl group). Crystals of a different space group (P 42_1_2 compared with P 3_1_21 for the previous ternary complex) were obtained, diffracting to a resolution of 2.0 Å and containing two Prim–PolC–DNA- bound complexes per asymmetric unit. The structure was solved by molecular replacement using the *Apo-*Prim–PolC structure (see ‘Methods’) and refined at 2.0-Å resolution (Table 1).

**Fig. 5:**
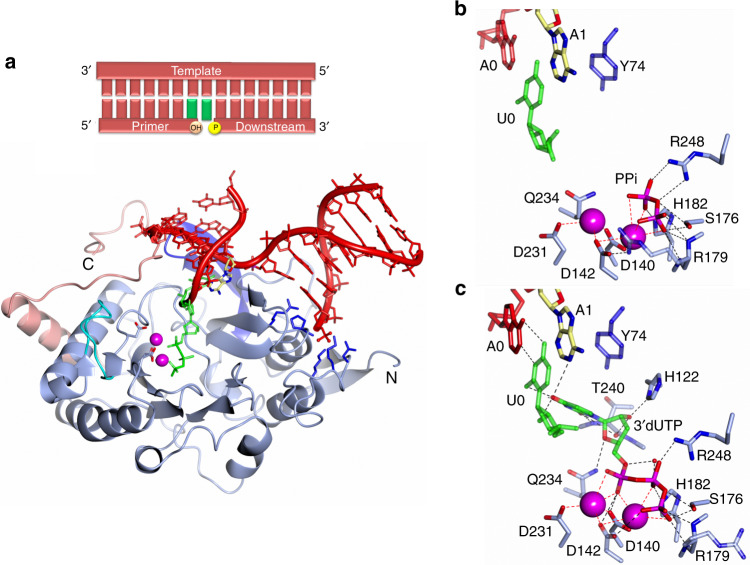
Crystal structures of post-catalytic Prim–PolC–DNA complexes. **a** Schematic of the 1-nt gapped post-turnover DNA substrate found in this pre-ternary post-catalytic complex (top panel). A ribbon-diagram representation of the post-catalytic Prim–PolC–DNA–NTP complex (bottom panel). The catalytic core is coloured sky blue, with the conserved loop structures from this family coloured dark blue, cyan and pink for Loops 1–3, respectively. The catalytic aspartate side chains are represented with the carboxylic oxygens shown in red, and bound manganese ions depicted as magenta spheres. Side chains involved in forming the 5′ phosphate-binding pocket are depicted in dark blue. Active- site geometries of the two forms of post-catalytic turnover Prim–PolC:DNA complex. **b** In this post-catalytic complex, nucleotidyl transfer has occurred, and inorganic phosphate (PPi) remains bound in the active site. The gate residue, R179, is in the closed conformation, preventing PPi from leaving. All the hydrogen bonds are depicted in black hatched lines, and dative bonding in red hatched lines. Catalytic manganese ions depicted as magenta spheres. **c** In this post-catalytic complex, PPi has left the active site, and R179 remains in the open conformation, allowing the binding of the next incoming 3′-dUTP in readiness for the next round of catalysis. Regions or side chains that have poor density are represented as transparent elements.

Comparing this structure with the previous *Apo* crystal structure (PDBID: 5Op0) revealed that the overall core fold of the protein is maintained, with a RMSD of 0.5 Å (over 323 Cα positions). The RMSD difference between the two ternary complexes is 1.49 Å (over 327 Cα positions). The structure revealed that the expected turnover reaction had occurred, as an incorporated ribonucleotide was now visible on the 3′ end of the primer strand, leaving a 1-nt gap. Further turnover was not possible due to the lack of a 3′-OH moiety on the newly extended primer strand. The active site contains two Mn^2+^ atoms and an incoming 3′-dUTP making W–C contacts with the templating base in the 1-nt gap (Fig. 5a).

The crystals contained two complexes in the asymmetric unit and, fortuitously, each of these complexes represents a different post-catalytic intermediate state. In one complex, the active site is occupied with inorganic pyrophosphate (PPi), the remnants of the previous nucleotidyl transfer reaction (Fig. 5b). PPi adopts the same conformation that it would have adopted as part of the triphosphate tail of an incoming NTP. This ‘snapshot’ illustrates the beginning of the final step of turnover on the reaction pathway in which the products diffuse away from the active site, defining the rate-limiting step for turnover^13^. Further examination of this complex shows the residues in contact with the PPi, and R179 is of particular interest as is it adopts multiple conformations within these structures (Fig. 5c). Here, R179 is wrapped around the γ phosphate and hydrogen bonds with a key catalytic residue, D142. R179 effectively creates a gate that is observed in the ‘closed’ position, thus preventing PPi from leaving the active site.

In the other complex in the asymmetric unit, we observed the next step in the enzymatic pathway, the binding of the next incoming rNTP into the active site, following PPi release (Fig. 5c). We define this as a post-catalytic pre-ternary complex as the NTP has not quite reached its ideal W–C templating position due in part to the disengagement of the upstream DNA, but its triphosphate tail is properly engaged in the active site. The ribose group of this NTP is almost bound as observed in the ternary complex, but a hydrogen bond with T240 is missing as the side chain faces in the wrong orientation, causing a less-stabilised binding. The R179 ‘gate’ is in the open position, presumably to allow PPi to leave the active site to facilitate the binding of the next incoming nucleotide (Fig. 5c).

### STD facilitates DNA gap-filling repair

**Fig. 6:**
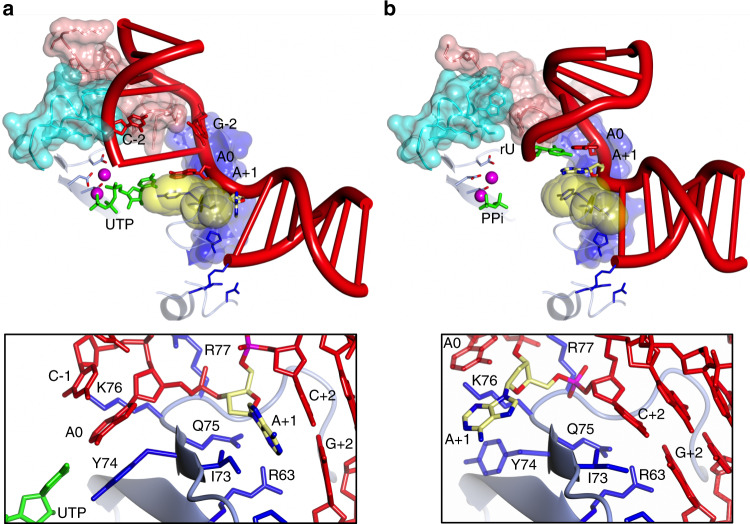
Synthesis-dependent template displacement (STD) influences DNA conformation through direct interactions between the gap and a hydrophobic wedge. **a** A schematic representation of the Prim–PolC/DNA/UTP ternary model emphasising how the splaying of the template strand and bases in the gap is influenced by a hydrophobic wedge (I73/Y74). Surface loops (1–3) also play key roles in orientating the upstream side of the gapped DNA. Loops 1–3 are represented as translucent surfaces coloured blue, salmon and cyan, respectively. The base pairing of the upstream DNA is disrupted at G/C-2 by interactions with Loop 3. The surface of I73/Y74 wedge is coloured yellow. The inset view (below) shows the side-chain interactions that participate in the splaying of the gap bases (A0 and A + 1) of the template strand. **b** A similar schematic representation of the post-turnover complex with PPi bound in the active site. This structure shows a significant difference in the bending angle and orientation of the upstream DNA following synthesis brought about by the change in splaying angle around the wedge and loss of contacts with Loops 2 and 3, induced by synthesis-dependent template displacement. The surface of I73/Y74 wedge is coloured yellow. The inset view (below) shows the relocation of the second gap base (A + 1) across the wedge into the active site following ribonucleotide incorporation opposite A0. Side chains with poor density are represented as transparent elements.

Comparison of the ternary and post-catalytic turnover complexes reveals that a remarkable transition occurs, as a result of STD, resulting in major changes in the orientation of DNA relative to the enzyme (Fig. 6a, b). The post-catalytic complex shows the conformation following a single turnover event, where the DNA has gone from a 2-nt to a 1-nt gapped product/substrate. The DNA has undergone a frameshift translation in the upstream direction, resulting in a loss of engagement of the upstream duplex with Loops 2 and 3. This disengagement is promoted by the increased splaying angle of the bases (111.6°) at the hydrophobic wedge I73/Y74, a difference of ∼14.3° compared with the pre-turnover complex. This increase in splay angle results in the template strand taking a path that leads away from the enzyme. In turn, the W–C-paired primer strand also has no contact with Loop 2, and the correct positioning of the incoming primer strand 3′-OH into the active site has yet to occur (Fig. 6a, b, lower inset panels). The energy liberated during phosphodiester bond formation by nucleotidyl transfer likely results in the primer strand being pushed upwards and displaced off Loop 3. Concomitantly, the unpaired base (A + 1) on the downstream side of the template strand is pulled over the hydrophobic wedge (I73/Y74), causing the position of the splayed DNA bases to change (from between A0/A + 1 and A + 1/C + 2 on the T strand). This conformation is stabilised by increased engagement with the templating strand around the gap position, interactions between K76, R77 and T/A0, which are not observed in the pre-turnover ternary complex. The splay angle of the bases is also influenced by the interactions of side chains from R77 and R97 (Loop 1) with T/A + 1. Loop 1 further stabilises the template strand through coordinating the phosphate from T/C-1 via interactions with P94, S95 and R97. The template strand is now almost disengaged from the protein, with non-bonded interactions between N321 (Loop 3), T/C-1 and T/G-2 (Loops 2 and 1, respectively). The template displacement caused by STD allows the downstream dsDNA interface (T/C + 2:D/G + 2) to sit flush on the N-terminal region of the enzyme. The axis of the downstream DNA duplex is now no longer offset and resembles the angles achieved in the reported binary/ternary Prim–PolD complexes (Fig. 6a, b; Supplementary Fig. 7). It is notable that the movement of the unpaired A + 1 base into the active site, previously held in an orphaned position on the downstream side of the wedge (I73/Y74), occurs following phosphodiester bond formation, but prior to the release of PPi, which resets the catalytic cycle (see below). Together, these structural intermediates support the existence of an STD mechanism that accommodates gaps that are larger than 1 nt in size, the assumption being that a 1-nt gap would sit on the enzyme with no distortions to the axis of the downstream DNA. For larger gaps, as observed with a 2-nt gap, STD is deployed, and the second unpaired base is accommodated in a position distal to the active site with a distortion to the axis of the downstream DNA.

## Discussion

Although significant progress has been made in our understanding of how DNA polymerases undertake repair synthesis, much less is known about how Prim–Pol family members bind to and extend DNA substrates. Prim–PolC belongs to the proper clade of archaeo-eukaryotic primases, which includes eukaryotic replicative primases (PriS/Prim1) and prokaryotic NHEJ-repair Prim–Pols (Prim–PolD)^2,14^. Currently, no structures of catalytically competent ternary complexes of either PriS or Prim–PolD have been reported, so little is known about how they catalyse primer extension. This study goes some way to addressing the paucity in our knowledge of these related catalytic mechanisms by providing the first structural glimpses of catalytic intermediates of a closely related Prim–Pol member in the act of binding to and extending a primer strand, in the context of a gapped DNA substrate.

Although members of the Prim–Pol superfamily share a number of common structural features, especially in their catalytic cores^14,15^, they have evolved distinctive functional adaptations that are requisite for their bespoke roles, including the acquisition of additional domains and structural elements. This is exemplified by Prim–PolD, whose prominent surface loops (Loops 1 and 2) facilitate DSB repair (Supplementary Fig. 8a). Loop 1 plays a key role in template strand positioning, particularly important for presenting 3′ overhanging termini to promote MMEJ^5,6^. Loop 2 also plays a prominent role in this process by promoting the acceptance of a 3′ end from incoming break termini to promote MMEJ and facilitate DSB-repair synthesis in *trans*. Although Prim–PolC retains similar loops and utilises them in analogous ways, there are clear differences in their specific roles as they operate in conjunction with an additional loop (Supplementary Fig. 8b), Loop 3, which is requisite for Prim–PolC’s bespoke roles in short gap-repair synthesis. To elaborate, Loop 3 makes a physical connection between Loops 1 and Loop 2, fixing these elements in place and occluding the space between them. In Prim–PolD, Loop 1 supports the template strand and directs it through this space (occupied by Loop 3 in Prim–PolC) so that it can act as an incoming primer in *trans*^5^. In Prim–PolC, Loop 1 gives minimal guidance to the template DNA strand, with most of the directing action on the template DNA coming from the splaying caused by I73/Y74. The upstream DNA duplex is supported by Loop 3 and held in place with the assistance of Loop 2, which supports the primer strand. Loop 3 stops the upstream duplex DNA from sliding back and forth via the locating pin of N321, which in conjunction with P320, inserts into and disrupts the base pairing at the −2 position. This tethering of the upstream duplex, in conjunction with the downstream duplex being bound via the 5′ phosphate, defines the action of Prim–PolC as a short patch gap-filling enzyme. Loop 3 also imparts a physical constraint on the maximum size of gap that can be accommodated, and its presence defines a subfamily of Prim–Pols that are specifically adapted to short gap filling in the context of excision repair.

**Fig. 7:**
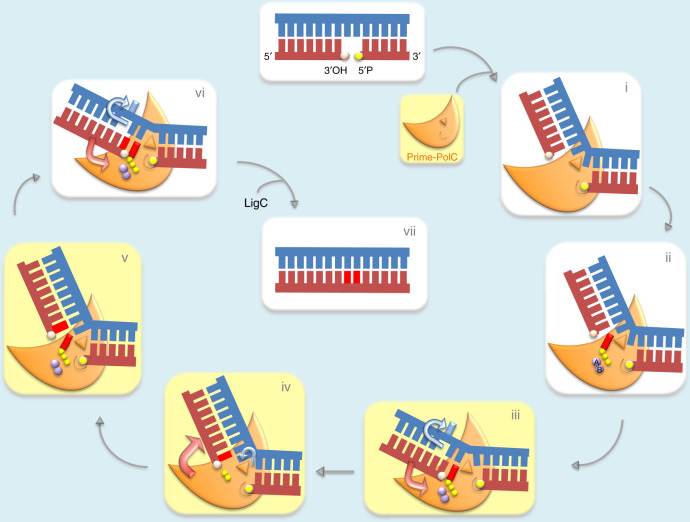
Proposed mechanism of gap-repair synthesis by Prim–PolC. Schematic diagram representing the elucidated Prim–PolC catalytic intermediates (yellow boxes) and their positions in the catalytic cycle. (i) Gapped DNA with a 3′-OH and 5′ phosphate is bound by apo Prim–PolC (PDBID: 5OP0). (ii) Upon binding, the 5′ phosphate is located in the binding pocket, and the hydrophobic wedge residues I73/Y74 splay the DNA via insertion between the unpaired bases. (iii) The binary complex then binds an NTP, the DNA splay angle decreases as the upstream DNA docks onto Loop 3 and is tethered via the insertion of the pin residue (N321) that, with P320, disrupts the base pairing at position −2 (PDBID: 6SA0). A process of Synthesis-dependent Template Displacement (STD) allows the ternary complex to be formed. (iv) Nucleotidyl transfer proceeds leaving PPi in the active site, and the extension process pulls the remaining unpaired base into the templating position. The extended upstream duplex disengages from Loop 3 and with an increase in splay angle (PDBID: 6SA1). (v) The gating residue (R179) moves into an open conformation, PPi leaves the active site and is replaced by the next incoming NTP. (vi) The DNA splay angle decreases as the upstream DNA docks onto Loop 3 and is tethered via the insertion of N321 that disrupts the base pairing at position −2. Another ternary complex is formed. (vii) Following nucleotidyl transfer, the upstream duplex disengages from Loop 3, and the downstream DNA disengages from the phosphate-binding pocket, leaving a nick that is then ligated by LigC to complete repair of the DNA.

The elucidation of structures of the key pre- and post-catalytic steps that describe Prim–PolC’s synthesis activities on gapped DNA substrates (Fig. 7) has uncovered a distinctive STD mechanism that keeps unpaired gap bases apart during repair synthesis, preventing the risk of both entering the active site. This mechanism operates by placing one of the unpaired bases of the gap into the active site and the other unpaired base(s) on the distal side, separated by a hydrophobic wedge of I73/Y74 (Fig. 7, steps ii and iii). In Prim–PolD, this wedge is formed by a pair of phenylalanines (F63/F64), and it is likely that the splayed DNA in this configuration is in a more stabilised conformation, due to the action of shielded hydrophobic side chains that likely prevent base displacement. However, in the case of Prim–PolC, the presence of an isoleucine residue appears to allow the distal base to slide over this wedge and into the active site (Fig. 7, step iv), but only after the previous base has been incorporated, driven by the considerable energy released during phosphodiester bond formation. The PPi-bound post-catalytic intermediate strongly supports the proposed STD mechanism as it suggests that base movement is a relatively rapid process. This template base relocation occurs concomitantly with bond formation and prior to the departure of inorganic pyrophosphate from the active site, which is requisite for the next round of nucleotide binding and incorporation.

A notable feature of both Prim–PolC–DNA complexes is the positioning of the unpaired bases of the gap. For the A + 1 base to move from the distal side to the proximal side of the wedge, there needs to be an element of sliding of the template strand over the hydrophobic wedge. Is this achieved by the ratcheting of the upstream strand upon extension, or is there disengagement of the DNA and rebinding? The current structures suggest an element of both models occurring. The enzyme holds onto the DNA substrate, via the 5′ phosphate on the downstream strand, but allows the upstream DNA to disengage from Loop 3 once synthesis has occurred (Fig. 7, steps v and vi). Presumably, the physical extension of the primer strand and frameshift movement of the upstream strand by STD is not prevented by N321. This movement has to happen, once the primer strand is extended, as there is no space left in the active site to accommodate the next gap-filling step. This motion allows a new incoming NTP to bind and, upon correct matching with the templating base, then the upstream strand will re-engage to ensure correct positioning of the primer strand (Fig. 7, steps v and vi).

The upper limit for gap filling is determined by the two-handed grip Prim–PolC exerts on gapped DNA, binding the downstream duplex via the 5′ phosphate and also the tethering of the upstream duplex in place in a synthesis-specific conformation (Fig. 7, steps iii and vi). Extension assays showed that a 3-nt gap can be accommodated but not a 5-nt gap. Although the STD mechanism can explain the handling of 2-nt gaps, how can Prim–PolC efficiently fill in 3-nt gaps? We previously reported that this family of enzymes has the ability to stabilise extra-helical or scrunched template bases with little distortion to the templating base^6^. Stabilising an extra-helical base, in conjunction with STD, would enable the enzyme to accommodate slightly longer gaps. However, gaps ≥4 nt pose a problem as there is a lack of room to accommodate all the unpaired bases, whilst still keeping the templating base in the correct position for base pairing with the incoming nucleotide. This is all in the context of the upstream DNA sitting on Loop 3 and being held in place by the locating pin of N321, in conjunction with P320, as well as the downstream DNA being held in place via the interaction of the 5′ phosphate with the binding pocket^16^.

A comparison of the mechanisms used by Prim–PolC and X-family polymerases to accommodate 2-nt gaps shows that they employ distinctive strategies. In the case of Pol lambda (*λ*), the unpaired template base of the gap is stabilised in an extra-helical conformation by a binding pocket formed by side chains from three amino acids^17^. This unpaired base does not influence the conformation of the downstream DNA, although it has been proposed that more unpaired bases would exert an influence similar to that observed in our current structure^17^. With Pol mu (*μ*), the unpaired base is accommodated in the active site, there is no scrunching as observed with Pol *λ* and the template base at the +1 position preferentially directs incorporation, giving a distinctive mutation signature during NHEJ^18^. Although Pol *λ* and *μ* function as NHEJ polymerases, they may also play some roles during BER^19,20^. In contrast, the STD mechanism of Prim–PolC prevents this overlapping function by restricting its substrate specificity, thus ensuring that this enzyme functions specifically in short gap-repair synthesis.

## Methods

### Purification of Prim–PolC proteins

Genes encoding Prim–PolC, were PCR-amplified using primers flanked with restriction digestion sites required for in-frame cloning into pET28 vector. Variant constructs of Prim–PolC were created by a site-directed mutagenesis protocol using overlapping primers. Variants of Prim–PolC constructed were T2 (Prim–PolC_1-312_), T3 (Prim–PolC_1-319_), Prim–PolC:P320G, Prim–PolC:N321A, Prim–PolC:N321G, Prim–PolC:K324A, Prim–PolC:I73F/Y74F and Prim–PolC:I73A/Y74A. All the proteins were designed to contain a N-terminal histidine tag. Proteins were purified according to routine laboratory procedures using ÄKTA purifier and compatible columns purchased from GE Healthcare. Briefly, pre-cleared cell lysates obtained after overexpression of recombinant proteins in *E. coli* Origami B pLysS strain were loaded onto Nickel Sepharose (Qiagen) column in Tris buffers [50 mM Tris, pH 8.0], washed extensively and eluted in a gradient of imidazole. Proteins were next loaded onto an ion-exchange column (Q-Sepharose, GE Healthcare) eluted in a gradient of NaCl and further purified on preparative gel-filtration columns (S200, GE Healthcare). The quality of proteins after each and every purification step was evaluated using sodium dodecyl sulfate (SDS) polyacrylamide gel electrophoresis (PAGE).

### Crystallisation and X-ray structure determination

The oligonucleotides used to generate the 2-nt gapped DNA for crystallisation were the following: T (5′-CGCTCGCAACGCACG-3′), P (5′-CGTGCG-3′) and 5′-phosphorylated D (5′-GCGAGCG-3′). T/P/D duplex DNA was prepared by mixing equal amounts of the oligonucleotides to give a final solution of 2 mM, then heating this solution to 95 °C and slowly annealing over 45 min to 4 °C in a PCR machine. Crystals of the Prim–PolC complexes were grown at 285 K by vapour diffusion as sitting drops. The Prim–PolC/DNA complex was prepared by mixing Prim–PolC and NTP at a ratio of 10:1 NTP:protein in the presence of 10 mM manganese chloride. The PolDom/NTP mix was then incubated with the DNA at a ratio of 300 µM protein to 600 µM DNA. The protein/NTP/DNA solution was screened at 0.5 μL mixed with 0.5 μL of crystallisation buffer (0.05 M sodium cacodylate (pH 6.5), 2 M ammonium sulfate and 0.01 M manganese chloride). Prior to data collection, crystals were soaked in mother liquor containing 20% ethylene glycol prior to snap freezing in liquid nitrogen. For the Prim–PolC/DNA/UpNHpp complex, X-ray diffraction data were collected at 100 K using a synchrotron source at station I03 Diamond Light Source, Didcot, UK. The diffraction data were processed with xia2^21^ with additional processing by programmes from the CCP4 suite^22^. The statistics for data processing are summarised in Table 1. Initial phases were obtained by molecular replacement with PHASER^23^ using Prim–PolC (5OP0) as a search model^16^. Iterative cycles of model building and refinement were performed using Coot^24^ and Phenix. A final refined model at 2.21-Å resolution, with an *R*_factor_ of 17.46% and *R*_free_ of 20.69%, was obtained. In all, 98.3% of residues are in preferred regions with 1.4% in allowed regions according to Ramachandran statistics. Structural images were prepared with CCP4mg^25^. The structure of this Prim–PolC ternary complex is deposited in the Protein Data Bank under accession code 6SA0.

For the Prim–PolC/DNA/3′-dUTP complex, X-ray diffraction data were collected at 100 K using a synchrotron source at station I04 Diamond Light Source, Didcot, UK. The diffraction data were processed with xia2^21^ with additional processing by programmes from the CCP4 suite^22^. The statistics for data processing are summarised in Table 1. Initial phases were obtained by molecular replacement with PHASER^23^ using Prim–PolC (5OP0) as a search model^16^. Iterative cycles of model building and refinement were performed using Coot^24^ and Phenix^12^. A final refined model at 2.01-Å resolution, with an *R*_factor_ of 18.45% and *R*_free_ of 20.42%, was obtained. In total, 98.9% of residues are in preferred regions with 0.9% in allowed regions according to Ramachandran statistics.

Due to the way the crystal was obtained (in situ turnover), there are some parts of the model that suffer from poor density due to multiple conformations and/or high *B* factors that indicate higher flexibility for these parts of the model. Nucleic acid elements G-2, C-3, A-4, C-5 and G-6 of the template strand, C + 8 and G + 9 of the downstream strand and G-1, C-2, G-3, T-4, G-5 and C-6 of the primer strand have poor fit to the density in the PPi complex (Supplementary Fig. 9a, b). Protein residues with poor fit to the density within this complex were Ser 4, Trp 219, Thr 240, Leu 301, Leu 313, Met 325, Pro 326, Gly 327, Glu 328, Pro 329, Pro 330, Val 332, Gln 333 and Pro 334 (Supplementary Fig. 9a, c, d). Nucleic acid elements A0, G-2, C-3 and A-4 of the template strand, G-3 and T-4 of the primer strand have poor fit to the density in the 3′-dUTP-bound complex. There was no density at all for positions upstream DNA at positions −5 and −6. Part of the bound 3′-dUTP also exhibits poor density fit (Supplementary Fig. 10a, b). Protein residues with poor fit to the density within this complex were Ser 4, Ala 5, Ala 6, Pro 79, Gln 80, Trp 219, Trp 220, Glu 222, Thr 240 and Pro 323–Lys 336 (Supplementary Fig. 10a, c, d). These residues are depicted as transparent elements when presented in the main figures. Structural images were prepared with CCP4mg^25^. The structure of this Prim–PolC chain-terminated ternary complex is deposited in the Protein Data Bank under accession code 6SA1.

### DNA gap-filling assay

DNA extension reaction mixtures contained 50 mM Tris-HCl (pH 7.5), 5 mM MgCl_2_, 100 μM MnCl_2_, 30 nM 6-FAM labelled DNA substrate, 250 μM NTPs and the indicated Prim–PolC mutant, in a total volume of 20 μl. After a set incubation time at 37 °C, reactions were terminated by adding stop buffer solution (95% (v/v) formamide, 0.09% (w/v) bromophenol blue and 20 mM EDTA). The resulting DNA extension products were resolved for 2 h at constant wattage of 20 W, on TBE-buffered 15% polyacrylamide gels containing 7 M urea. Detection of fluorescently labelled oligonucleotide products was carried out using Fujifilm FLA-5100 fluorescent image scanner. The contrast on all these gel-based images used in subsequent figures was adjusted in the linear range. Uncropped gels used to produce these figures are shown in Supplementary Fig. 11.

### 2-Aminopurine-displacement assay

Emission fluorescence data for 2-aminopurine (2-AP) DNA structures (2-AP at primer −2 position) in the presence of Prim–PolC were collected on 96-well plate (black nuc96) with CLARIOstar—BMG Labtech plate reader. Samples were excited at 320+/−10 nm, and fluorescence emission data scans were collected at 370+/−20 nm. Dichroic mirror was set to 342.5 nm. In all, 100-µl reaction containing 1 µM 2-AP-labelled DNA substrate, 0–1 µM Prim–PolC, 50 mM, +/−250 µM ATP, 50 mM Tris (pH 7.5), 25 mM NaCl and 5 mM CaCl_2_ (or a mixture of CaCl_2_ and MgCl_2_) was inoculated for 10 min at room temperature before data collection at 25 °C. The background (reaction without protein) was subtracted from 2-AP DNA fluorescence. All data were related to 1 µM positive control substrate that had 100% of 2-AP unpaired (oKZ282 + oKZ283: overhang with primer containing 2-AP on −2 position and template with the abasic site at −2 position (opposite to 2-AP)).

### DNA primers and substrates

T2 (Prim–PolC1-312):

CGGGGCTAAGGTGATCTGCCCTACCCGCCGAACTA Fwd

GATCACCTTAGCCCCGCTCCTCGTCGGCGGCG Rev

T3 (Prim–PolC1-319):

CTACCCGTAACCGAACTACCCGAAGATGCCCGGC Fwd

AGTTCGGTTACGGGTAGGGCAGATCACCGAGGCC Rev

Prim–PolC:P320G:

GGTGATCTGCCCTACCCGGGCAACTACCCGAAGATGCCC Fwd

GGGCATCTTCGGGTAGTTGCCCGGGTAGGGCAGATCACC Rev

Prim–PolC:N321A:

ACCCGCCGGCGTACCCGAAGATGCCCGGCGAA Fwd

GTACGCCGGCGGGTAGGGCAGATCACCGAGGC Rev

Prim–PolC:N321G:

GATCTGCCCTACCCGCCGGGCTACCCGAAGATGCCCGG Fwd

CGGGCATCTTCGGGTAGCCCGGCGGGTAGGGCAGATC Rev

Prim–PolC:K324A:

CTACCCGGCGATGCCCGGCGAACCGCCACGG Fwd

GGCATCGCCGGGTAGTTCGGCGGGTAGGGCAGAT Rev

Prim–PolC:I73F/Y74F:

GCGAGGAGTTCTTCCAGAAACGGGTGCCGCAGAAGCAT Fwd

CTGGAAGAACTCCTCGCCCTCGATGCCGTCGGGGAA Rev

Prim–PolC:I73A/Y74A:

GCATCGAGGGCGAGGAGGCCGCCCAGAAACGGGTGCCGCAG Fwd

CTGCGGCACCCGTTTCTGGGCGGCCTCCTCGCCCTCGATGC Rev

oKZ282: 5′-CTATGAGCGAATCG**2AP**C

oKZ279: 5′-AGTCGCATAGTGTAGTCATG**T**CGATTCGCCATAG

1-nt gap: P-5′-CGACTACACTATGCGACT

2-ng gap: P-5′-GACTACACTATGCGACT

3-nt gap: P-5′-ACTACACTATGCGACT

5-nt gap: P-5′-TACACTATGCGACT

oKZ283: 5′-AGTCGCATAGTGTAGTCATG**X**CGATTCGCTCATAG (**X** = abasic site)

Positive control: oKZ282 + oKZ283

Overhang: oKZ282 + oKZ279

Gap substrates: oKZ282 + oKZ279 + x-nt gap

### Protein thermal shift assay

In all, 100-μl reaction contained 1 μM protein (Prim–PolC), 5× SYPRO Orange (10,000× stock solution from Invitrogen), 50 mM Tris, pH 7.5 and 250 mM NaCl. Reaction without protein was used as a control. In total, 25 μl of each sample was aliquoted in triplicate into a 96-well plate and sealed with sealing foil. Protein-melting experiments were carried out using the LightCycler 480 System II (Roche). The fluorescence was measured every 0.05 °C increment with excitation 465 nm and emission 580 nm. Denaturation curve fluorescent signal was acquired within a range of 20–99 °C using a ramping rate of 0.03 °C s^−1^. To calculate the melting temperature (**T**_m_), the Roche Protein Melt Tool was used, using the first-derivative method, as described by the manufacturer.

### Reporting summary

Further information on research design is available in the Nature Research Reporting Summary linked to this article.

## Supplementary information

Supplementary Information

Reporting Summary

## Data Availability

The data that support this study are provided in full in the ‘Results’ section and the Supplementary Information accompanying this paper, and are available from the corresponding author upon reasonable request. The crystal structures have been deposited in the Protein Data Bank and can be accessed using accession codes 6SA0 and 6SA1.
