# Supplementary material for: Molecular basis for DNA repair synthesis on short gaps by mycobacterial Primase-Polymerase C
Source: Nat Commun. 2020 Aug 21;11:4196. doi: 10.1038/s41467-020-18012-8 (PMC7442782; doi:10.1038/s41467-020-18012-8)
Supplement: Supplementary file 1 — Supplementary Information [file 41467_2020_18012_MOESM1_ESM.pdf]

Supplementary information

Synthesis-dependent template dislocation facilitates nucleotide incorporation during DNA gap repair

Brissett et al.

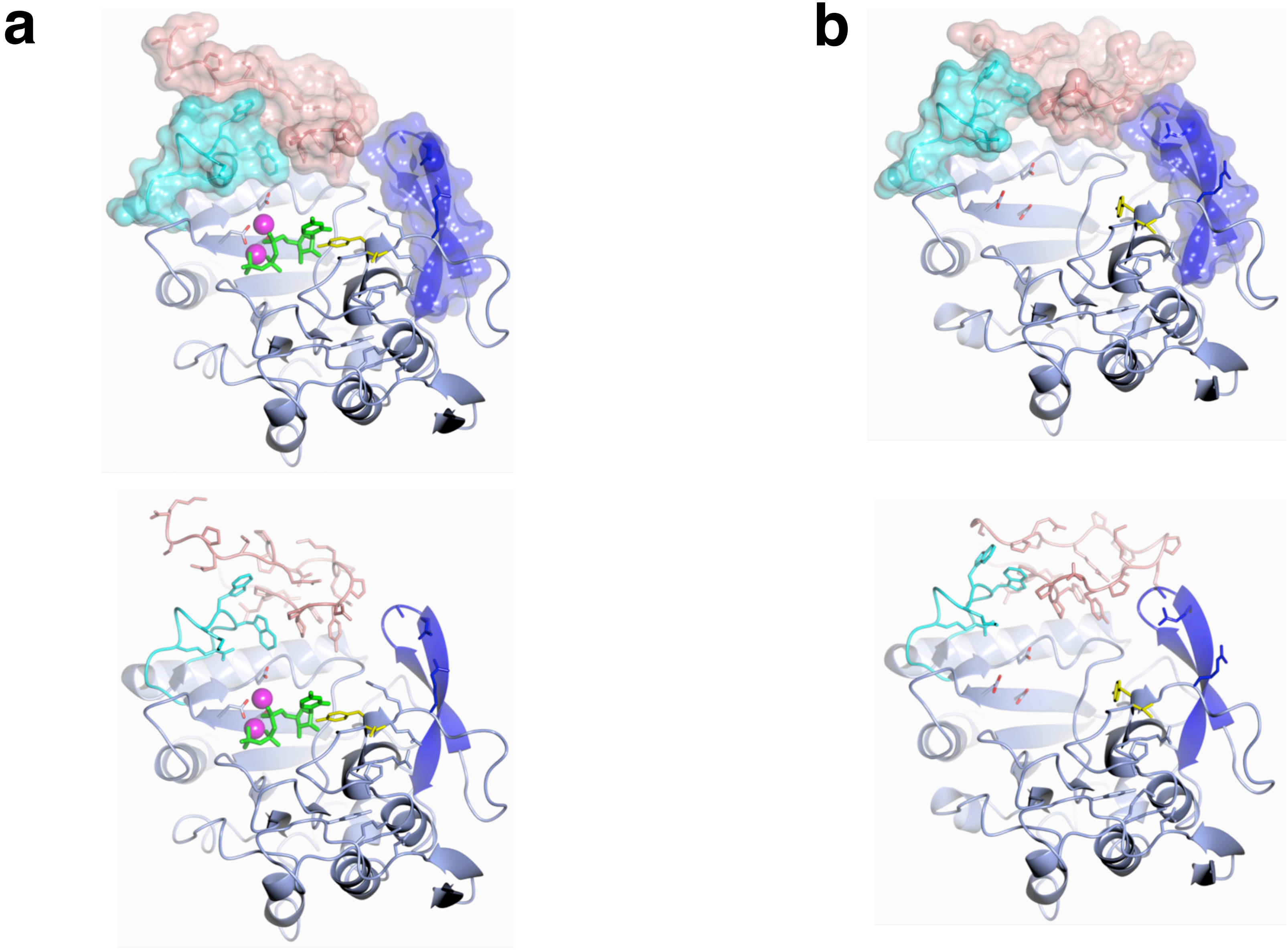

Supplementary Figure 1. Comparison of the Prim-PolC apo- & ternary complexes
(a) Schematic ribbon representations of the Prim-PolC ternary complex. The DNA is omitted for clarity. **(b)** The apo structure of Prim-PolC (PDBID: 5Op0) in the same orientation as **(a)**. The structures are depicted with (top panels) or without (bottom panels) surface accessible features covering Loops 1 (Blue), 2 (cyan) and 3 (salmon). The nucleotide is shown in green and metal ions in magenta.

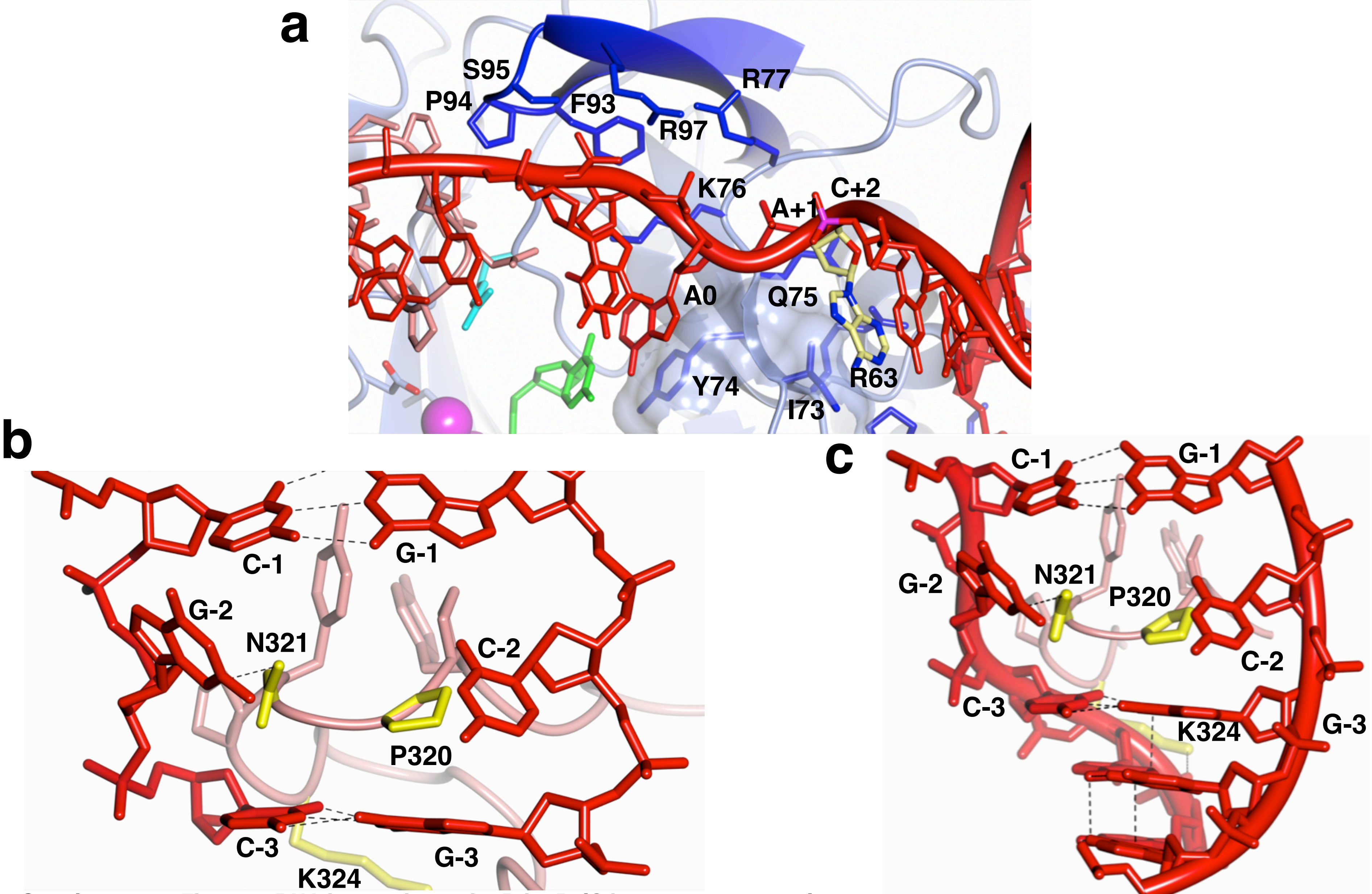

Supplementary Figure 2. DNA interactions with Prim-PolC in the ternary complex

Ribbon representations of the specific DNA interactions with Prim-PolC. **(a)** The molecular wedge of I73 / Y74 splaying the template DNA strand resulting in a significant kink along at this point in its axis. The solvent accessible surface is depicted to show the surface available for supporting DNA. Additional residues that contact the template DNA are also depicted. The catalytic manganese ions are coloured magenta and the UpNHpp is cyan. Protein and loop elements are coloured as previously described. **(c & b)** Two views showing the molecular interactions of Loop 3 with the upstream region of the bound ds DNA. Key interacting residues mutated in this study are shown in yellow.

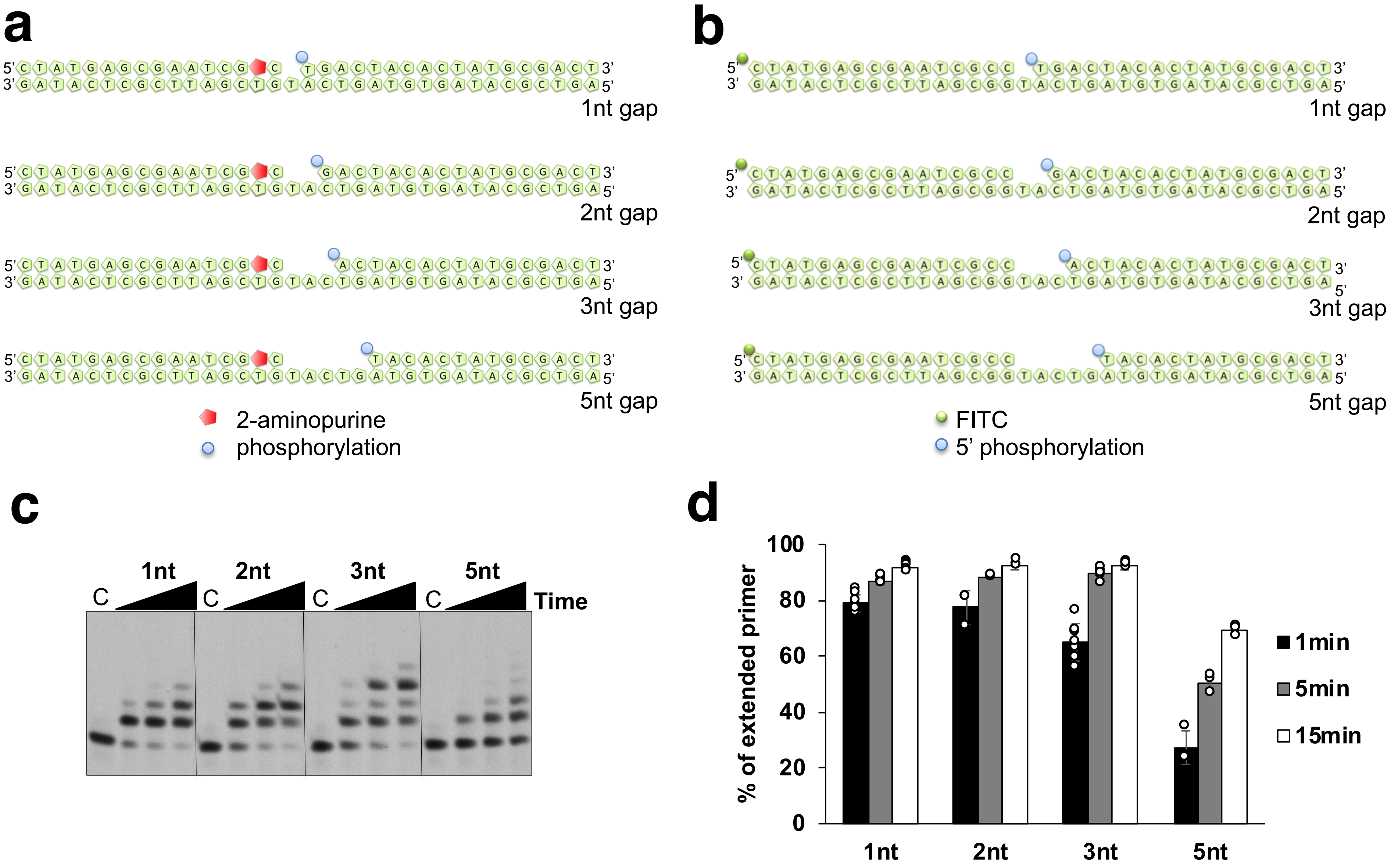

Supplementary Figure 3. Prim-PolC fills in short gaps on DNA.
(a) Schematic representations of substrates used in 2-aminopurine assays and **(b)** substrates used in gap-filling assays. **(c)** Gap-filling activity of Prim-PolC on different substrates. In primer extension assays, 30 nM of 5′-fluorescein labelled 36-mer containing a different single-stranded gaps, with phosphorylation of the 5′-end of the gap, was extended by Prim-PolC (300 nM) in the presence of a 250 µM rNTPs mix for 1, 5,15 min at 37 °C. Control (C) lane contains no protein. **(d)** Quantification of Prim-PolC primer extension on different substrates. Data shown are representative of the mean of at least three individual experiments and error bars show the standard deviation and the dot plots show corresponding data points.

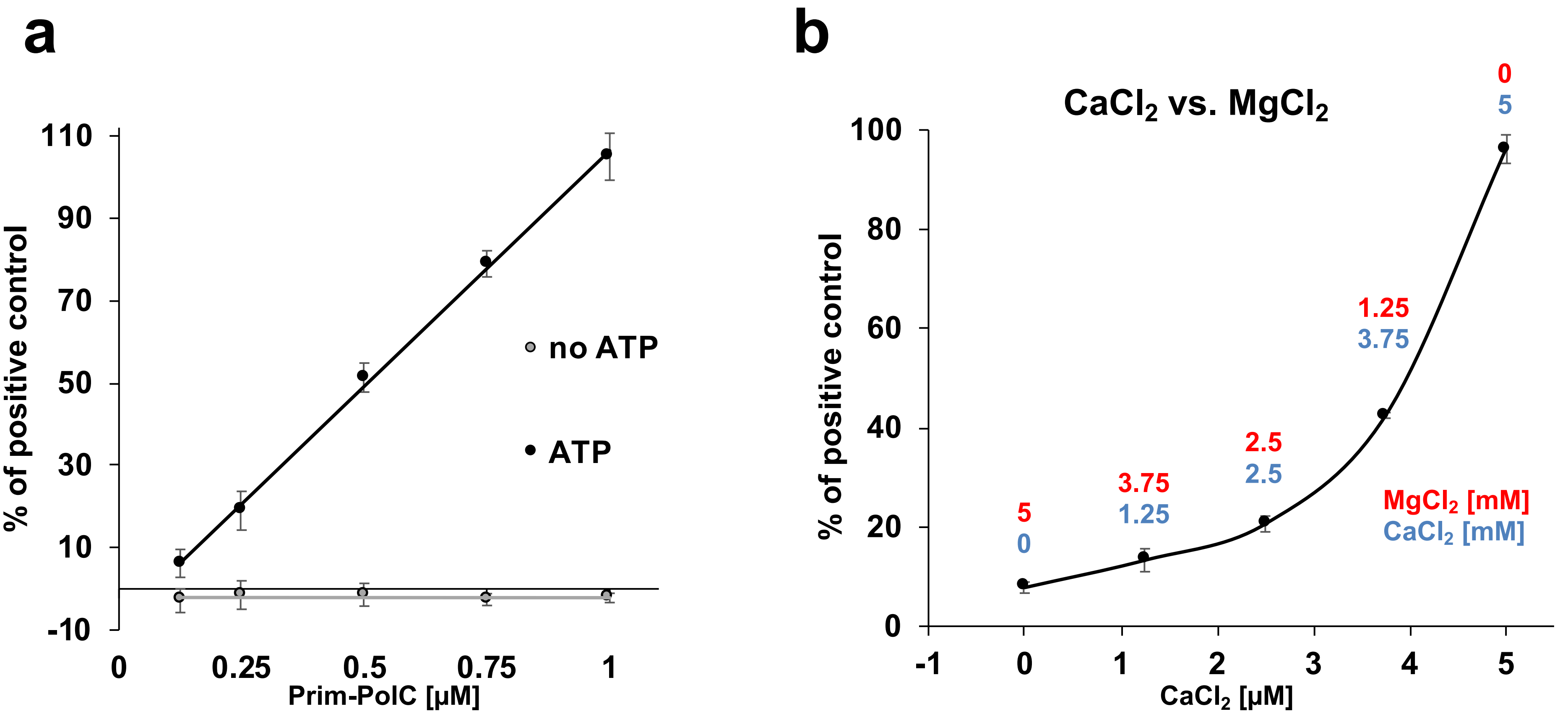

Supplementary Figure 4. (a) Effect of ATP on Prim-PolC base flipping. 2-aminopurine assay: Quantification of primer -2 base flipping by Prim-PolC on a 2nt gap substrate in presence of Ca^{2+} ions, with and without 250μM ATP. **(b)** Effect of different ions on base flipping. 2-aminopurine assay. Quantification of primer -2 base flipping by Prim-PolC on a 1nt gap substrate in presence of 250μM ATP and Ca^{2+} and / or Mg^{2+} ions. Data shown are representative of the mean of at least three individual experiments and error bars show the standard deviation.

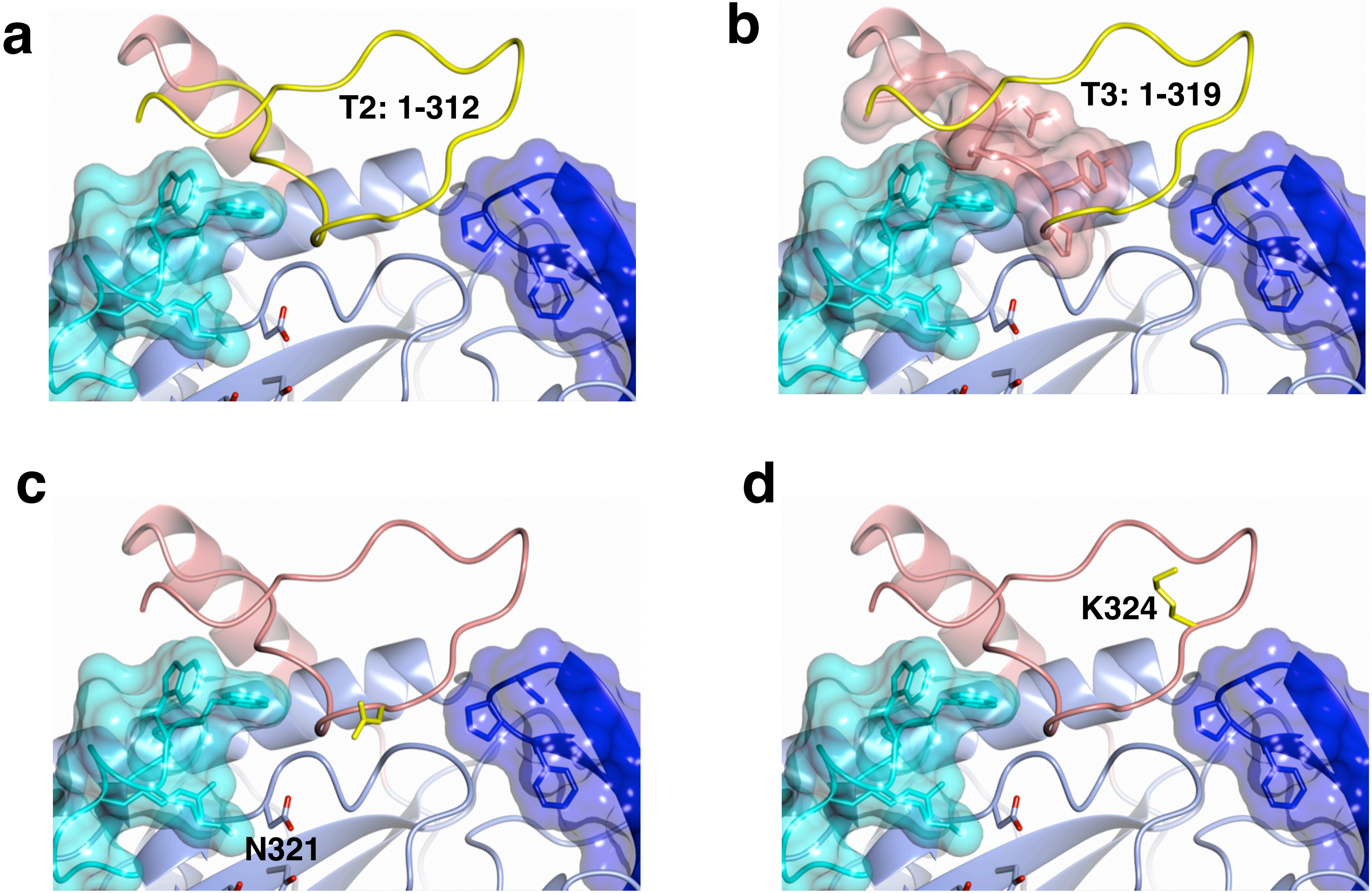

Supplementary Figure 5. Graphical representations of mutated regions of Loop 3.
Schematic ribbon representations of Prim-PolC with portions of the structure highlighting the region to mutated. **(a)** Model of T2 (Prim-PolC$_{1-312}$) with residues 313 to the end coloured yellow. **(b)** Model representation of T3 (Prim-PolC$_{1-319}$) with residues 313 to the end coloured yellow. **(c)** Model representation of Prim-PolC with N321 highlighted in yellow. **(d)** Model representation of Prim-PolC with K324 highlighted in yellow.

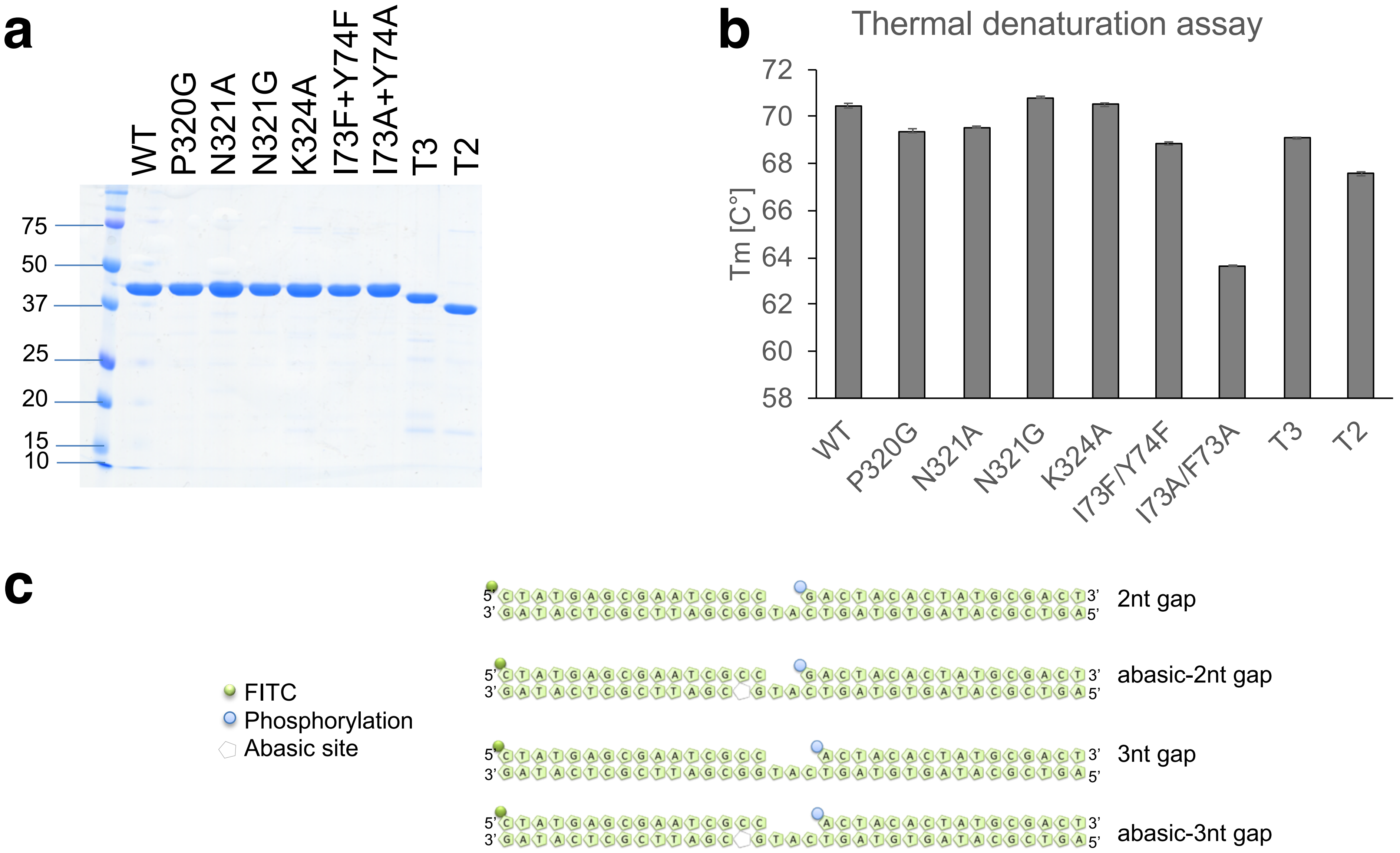

Supplementary Figure 6. Quality and thermal stability of Prim-PolC mutants. (a) Prim-PolC on SDS-PAGE. 50pmol of *E. coli* expressed and purified Prim-PolC mutants were resolved on 12% SDS-page and Coomassie stained. **(b)** Thermal stability of Prim-PolC. 100 μl reaction contained 1 μM protein (Prim-PolC), 5x SYPRO Orange, 50mM Tris, pH 7.5 and 250mM NaCl. Denaturation curve fluorescent signals were acquired within a range of 20–99°C, with excitation at 465nm and emission 580nm. Data shown are representative of the mean of at least three individual experiments and error bars show the standard deviation. **(c)** Gap-filling activity of Prim-PolC / WT on (template -2 position) abasic 3nt gap substrate. In primer extension assays, 30 nM of 5′-fluorescein labelled 36-mer containing a different single-stranded gaps, with phosphorylation of the 5′-end was extended by Prim-PolC (30 nM) in the presence of a 250 μM rNTPs mix for 1, 5,15 min at 37 °C.

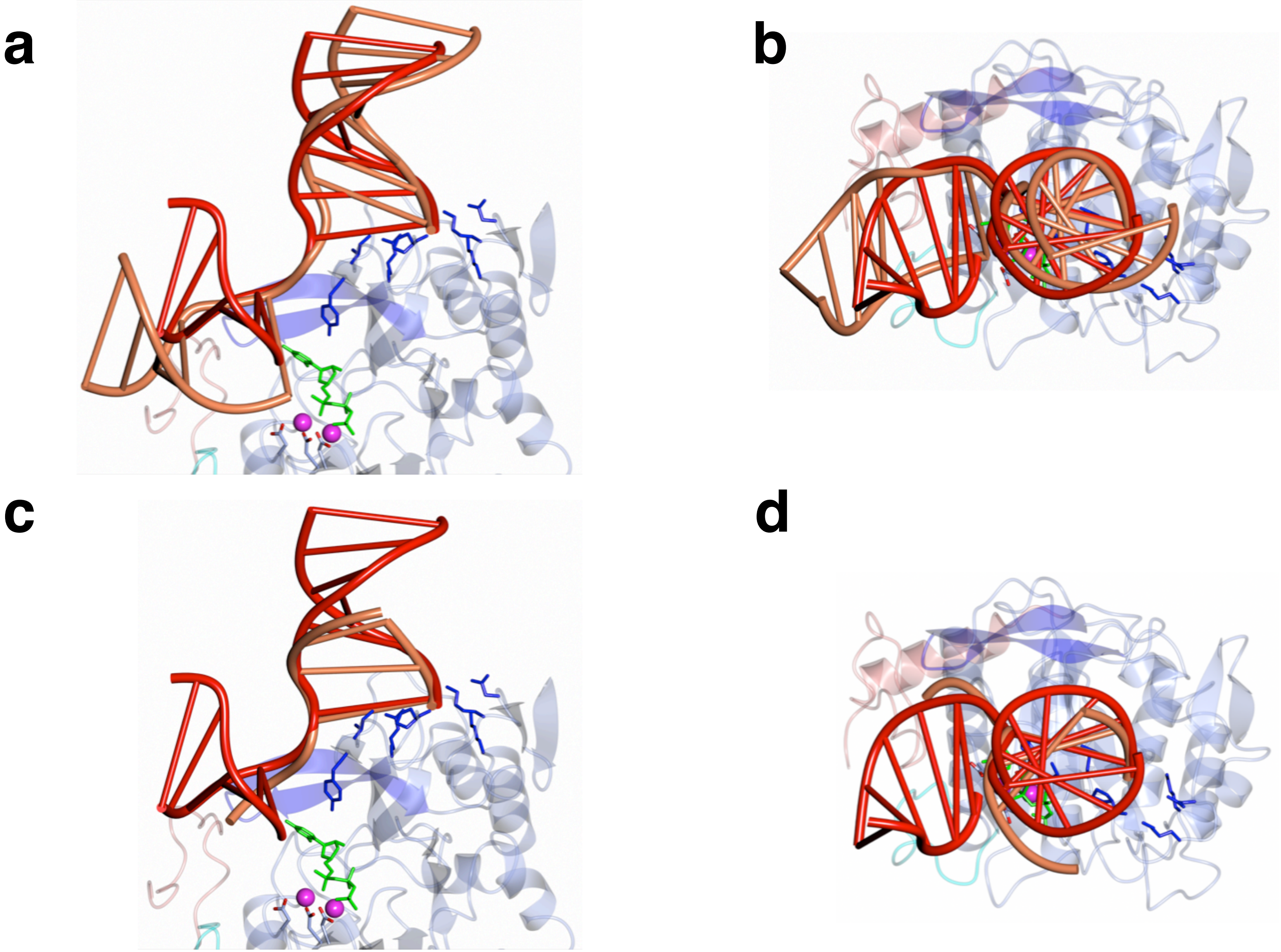

Supplementary Figure 7. Effect of <u>S</u>ynthesis-dependent <u>T</u>emplate <u>D</u>isplacement (STD) on DNA axis orientation.
(a) Schematic ribbon representations of the current post-catalytic complex superposed with DNA derived from differing Prim-Pol structures. The DNA from the ternary complex (coral) is superposed onto the post-catalytic complex. **(b)** Top view of the model in (a). **(c)** DNA from the Prim-PolD / gapped DNA complex (PDBID: 4MKY) is superposed onto the post-catalytic complex. DNA from the Prim-PolD complex is coral. The post-catalytic complex is coloured as previously described. **(d)** Top view of the model in (c).

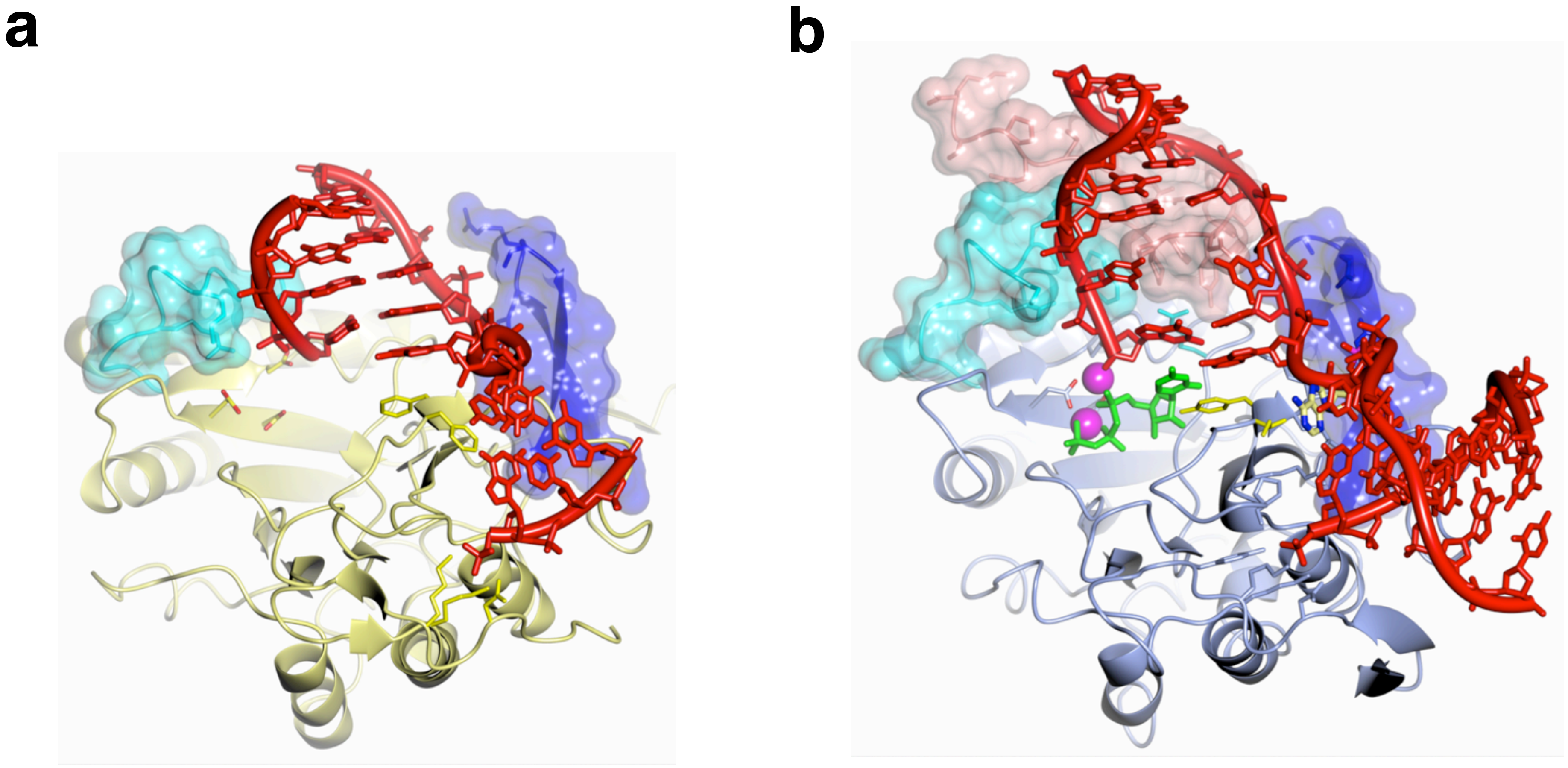

Supplementary Figure 8. Comparison of Prim-PolC & Prim-PolD DNA complexes
(a) Schematic ribbon representations of the Prim-PolD / gapped DNA complex (PDBID: 4MKY) in the same orientation as **(b)** the current ternary Prim-PolC complex (PDBID: 6SA0). Colour scheme as described in previous figures and Loops 1-3 depicted with surface accessible surfaces. Loop 1 depicted in blue, Loop 2 in cyan and Loop 3 in salmon pink.

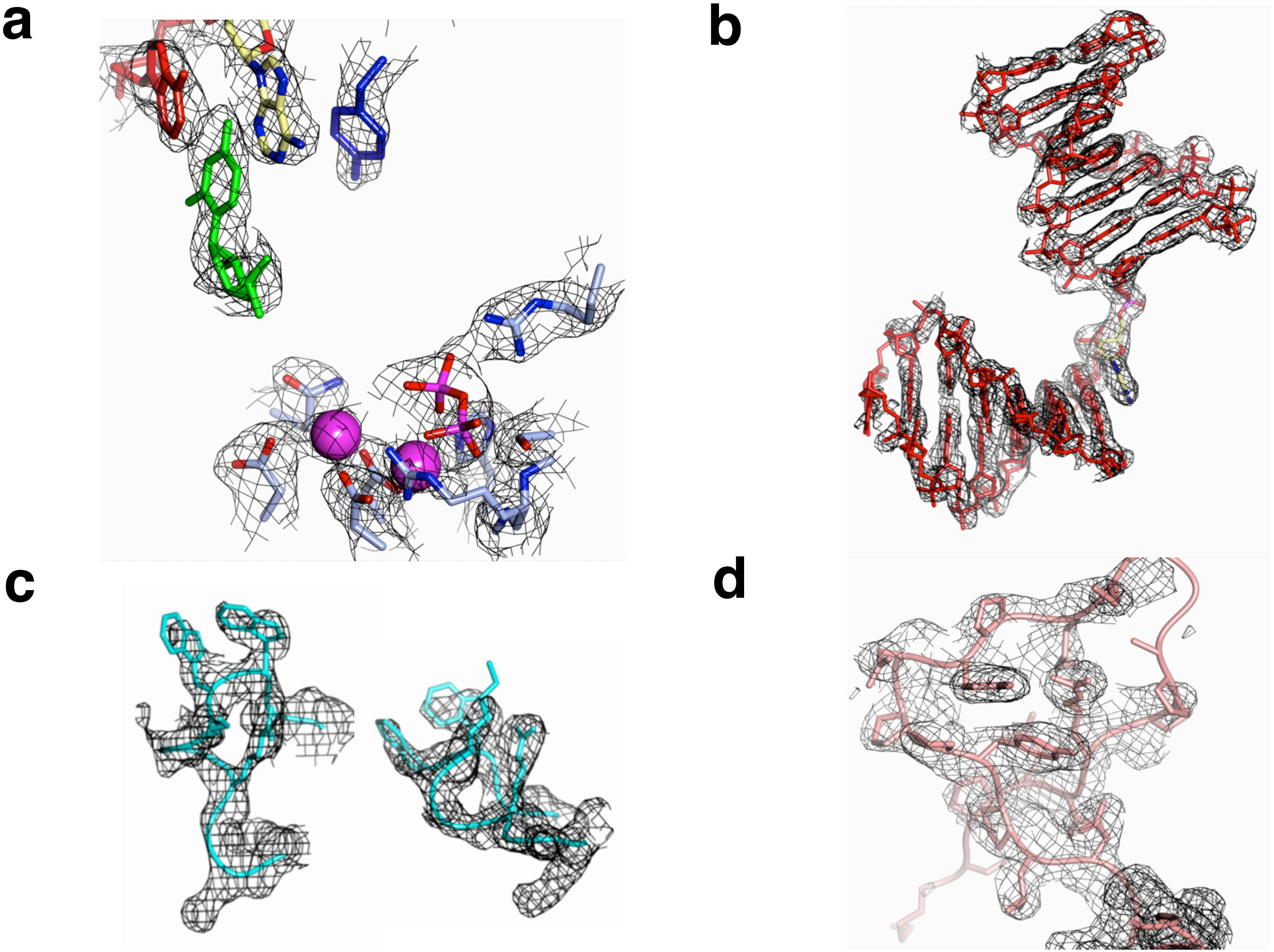

Supplementary Figure 9. Composite omit maps of key elements of the post-catalytic Prim-PolC–DNA complex with PPi bound in the active site. Schematic representations of the Prim-PolC ternary complex. **(a)** The active site of the structure showing PPi bound next to the metal ions (magenta). **(b)** DNA depicted in isolation from the same structure. **(c)** Loop 2 in two orientations. **(d)** Loop 3. Composite omit maps (black) at a contour level of 1 sigma.

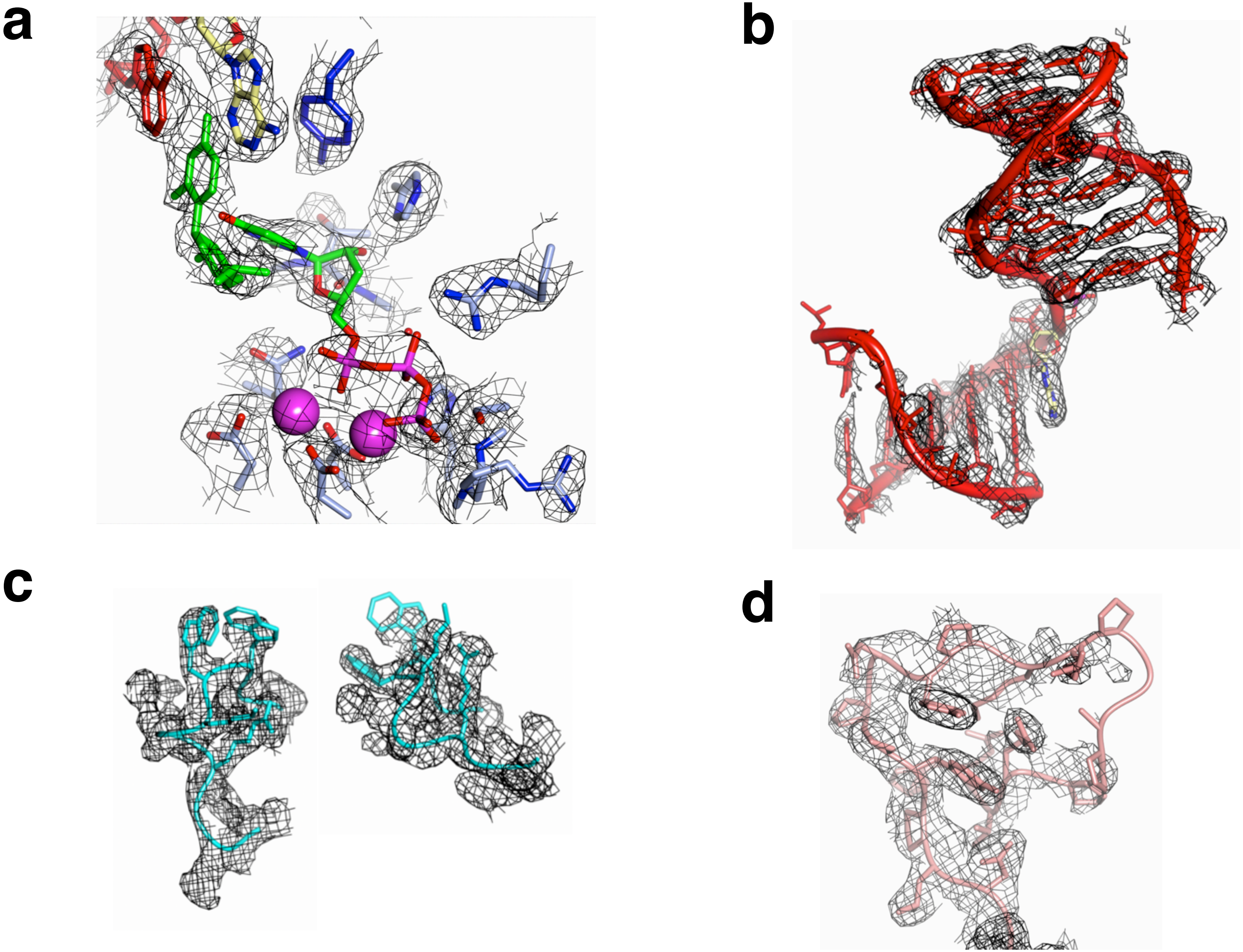

Supplementary Figure 10. Composite omit maps of key elements of the post-catalytic Prim-PolC–DNA complex with an incoming 3’-dUTP bound in the active site. **(a)** The active site of this structure containing the incoming 3’-dUTP and metal ions (magenta). **(b)** DNA depicted in isolation from the same structure. **(c)** Loop 2 in two orientations. **(d)** Loop 3. Composite omit maps (black) at a contour level of 1 sigma.

Figure 4a

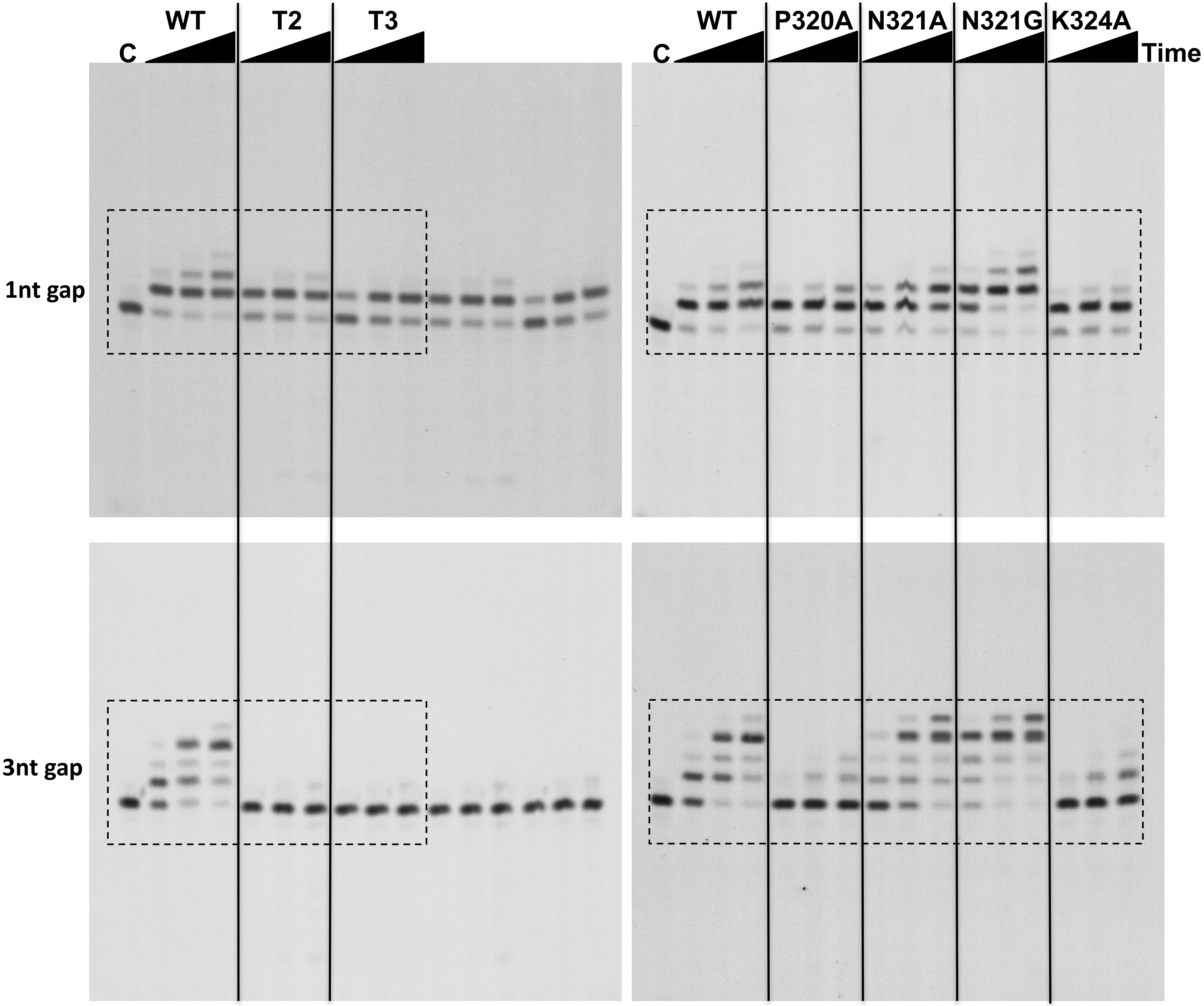

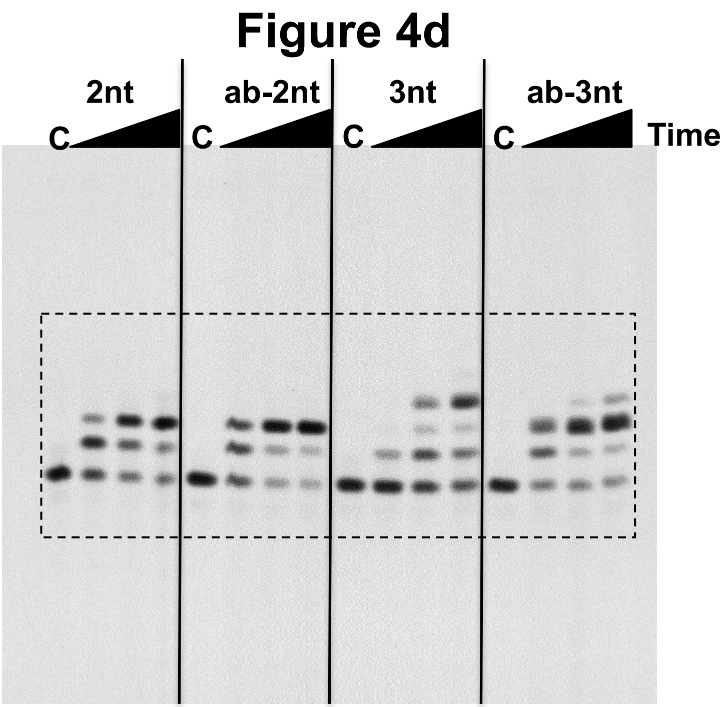

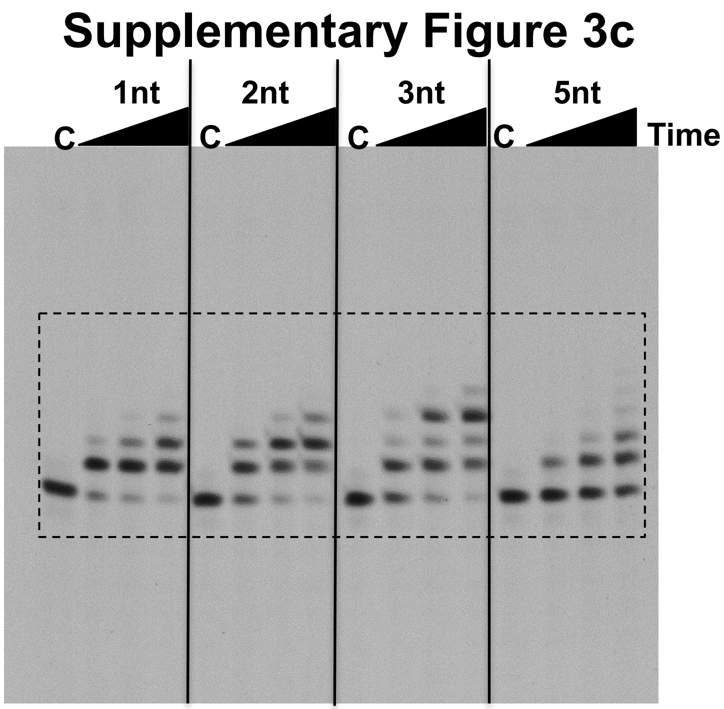

Supplementary Figure 11. Images of the uncropped gel-based fluorescence data used in this study. Cropped regions from these gels used in the presented figures are shown as hatched boxes.
